# Recent Advances in Development of Waste-Based Polymer Materials: A Review

**DOI:** 10.3390/polym14051050

**Published:** 2022-03-06

**Authors:** Krzysztof Formela, Maria Kurańska, Mateusz Barczewski

**Affiliations:** 1 Department of Polymer Technology, Faculty of Chemistry, Gdańsk University of Technology, Gabriela, Narutowicza 11/12, 80-233 Gdańsk, Poland; 2 Advanced Materials Center, Gdańsk University of Technology, Gabriela Narutowicza 11/12, 80-233 Gdańsk, Poland; 3 Department of Chemistry and Technology of Polymers, Cracow University of Technology, Warszawska 24, 31-155 Kraków, Poland; maria.kuranska@pk.edu.pl; 4 Faculty of Mechanical Engineering, Institute of Materials Technology, Poznan University of Technology, Piotrowo 3, 61-138 Poznan, Poland; mateusz.barczewski@put.poznan.pl

**Keywords:** polymers, blends, composites, sustainable development, manufacturing, recycling, case study of Poland

## Abstract

Limited petroleum sources, suitable law regulations, and higher awareness within society has caused sustainable development of manufacturing and recycling of polymer blends and composites to be gaining increasing attention. This work aims to report recent advances in the manufacturing of environmentally friendly and low-cost polymer materials based on post-production and post-consumer wastes. Sustainable development of three groups of materials: wood polymer composites, polyurethane foams, and rubber recycling products were comprehensively described. Special attention was focused on examples of industrially applicable technologies developed in Poland over the last five years. Moreover, current trends and limitations in the future “green” development of waste-based polymer materials were also discussed.

## 1. Introduction

Polymers have specific properties, such as e.g., high strength, light-weight, toughness, resistance to corrosion, relatively easy processing, and low-cost production, resulting in polymer-based blends and composites to be commonly applied in different branches of industry [1,2,3,4,5].

Consequently, the development of plastics and elastomers technologies is observed each year. Recent statistical data show that during the last 10 years, global production of plastics has grown by ~37%, which is equal to an increase at a level of around 100 million metric tons of polymeric materials. Financial data indicate that the global plastic market size in 2020 is worth approximately 579.7 billion US dollars. This indicates a value growth of ~15.5% within the last five years. According to estimations, in 2028, the value of the global plastic market should reach 750.1 billion US dollars, with a compound annual growth rate of 3.4% between 2021 and 2028 [6].

In 2019, the global production of plastics reached 368 million metric tons, from which only 57.9 million metric tons were produced in the European Union [7]. However, it is worth mentioning that in 2020, global production decreased by 0.3%, which is related to obvious limitations caused by the COVID-19 pandemic. The further consequences of this decreasing trend are currently unknown, however it seems that after global economy stabilization, the increasing trend in plastics production is expected to continue.

In Europe, the main demand for plastics is generated by six countries: Germany (24.2%), Italy (13.8%), France (9.5%), Spain (7.8%), United Kingdom (7.1%), and Poland (7.0%), which takes almost 70% of the EU market of plastics [8]. Considering plastics demand by their application fields, it can be categorized into: packaging (39.6%), building and constructions (20.4%), automotive (9.6%), electrical and electronic (6.2%), household, leisure, and sport (4.1%), agriculture (3.4%), and others (16.7%).

The increasing demand for the global production of plastics resulted in a higher amount of post-production and post-consumers wastes. Therefore, economic management and further recycling of a large volume stream of waste plastics is still a serious environmental problem.

According to Plastics Europe, in 2018, around 29.1 million tons of waste plastics were collected in European countries, which subsequently were managed by: landfilling (24.9%), recycling (32.5%), or energy recovering (42.6%). In Poland, plastics recycling and energy recovery levels are equal to 27.5% and 30.3%, which are considerably below average for EU countries.

The common solution for this burning issue was the export of waste plastics and their management by other countries in the past. However, from 2016 to 2018, waste plastics exports outside EU countries decreased by 39%, which was related to an announcement by China that imposed an unprecedented ban on its import of most plastic waste from 2018 [9]. As expected, this caused a sharp decline in global plastic waste trade flow [10] and forced European countries to research and develop new strategies regarding waste plastics and rubber utilization.

The first step in plastics and rubber recycling is the suitable classification, separation, and washing (when necessary) of waste materials. Żenkiewicz and Żuk [11,12] comprehensively reviewed the possibility of applying of tribocharging and electrostatic for the separation of polymers. Moreover, the authors studies highlighted important problems in separating biodegradable polymers from non-biodegradable materials [13,14], which should increase in the near future.

**Figure 1:**
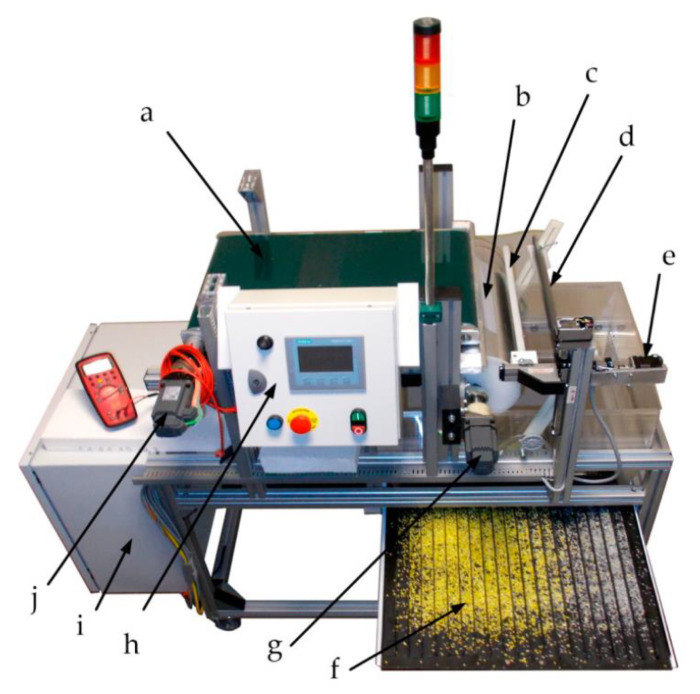
The electrostatic separator developed by research group from the Poznan University of Technology: a—feeder; b—drum; c—corona electrode; d—deflecting electrode; e—electrode positioning system; f—collector of material; g—drum drive; h—HMI control panel; i—control cabinet; and j—feeder drive [16].

The research works in this field were recently continued by the Czarnecka-Komorowska group from Poznan University of Technology, which investigated the tribo-electrostatic separation technique for cable waste from the automotive industry [15] or mixtures of poly(ethylene terephthalate) and high-density polyethylene [16]. The electrostatic separator designed and constructed by the authors is presented in Figure 1.

The results showed that the discussed electrostatic separator is a promising and useful tool for the circular economy of waste plastics.

The second step of waste plastics and rubber recycling is grinding or granulation, which allows for a suitable size reduction that is necessary for further processing (e.g., extrusion, injection molding) [17]. As presented by Al-Zubiedy [18], the dimensionally stable precision grinding design should include the following aspects: (i) polymer characteristics (structure and properties) and (ii) granulation/grinding method (construction, tools, and machines) and conditions (mixing, shearing, temperature). The combination of the above-mentioned factors affects the disintegration mechanisms of waste plastics and rubber and consequently features of obtained products, such as particle size distribution, average particle size, or surface morphology [19].

Flizikowski, Wełnowski, and Dudziak [20,21] designed and constructed supersonic aero-kinetic mill and studied the impact of grinding conditions on the size reduction of montmorillonite–nanofiller commonly used in polymer composites, e.g., as flame retardant additive [22].

Spina and Cavalcante [23] evaluated the grinding of unfilled and glass fiber reinforced polyamide 66. The results showed that the presence of glass fibers resulted in lower surface quality of ground polyamide 66. The authors pointed to the necessity of future investigations of the correlation between temperature profile and exchanged forces between material and grinding tool, which allows a better understanding of the process.

Hoyer, Kroll, and Sykutera [24] compared the cryogenic grinding of waste tire rubber (passenger car and truck tires) technology with different ambient grinding processes, such as solid-state shear extrusion; cracker mill; wet grinding; or hyperboloidal cutting mill. The summary of results obtained for truck tires is presented in Table 1. The cryogenic grinding technique has the highest throughput (588 kg/h) among the analyzed technologies (in the range of: 1.2–160 kg/h) for studied grinding methods and parameters. Considering energy consumption, the lowest values were determined for wet grinding (525 Wh/kg), while the highest for solid state shear extrusion (3132 Wh/kg).

Many scientific reports about grinding and granulation of waste polymers and rubber are still rather limited compared to other processing methods. Research about mechanochemistry in waste management [25] combined with novel technologies related to the synthesis of modified or hybrid fillers [26,27] or polymeric materials dedicated for special applications (e.g., polymeric powders dedicated for selective laser sintering 3D printing) indicate further development of grinding and granulation technologies in the coming years (especially those working in a continuous manner).

The report entitled “Sustainable Plastics Strategy” was recently published in the SUSCHEM—European technology platform for sustainable chemistry [28]. The main approaches of sustainable development of plastics proposed by SUSCHEM are summarized in Table 2.

This review work presents recent advances in the field of waste-based polymer materials. Considering approaches of “Sustainable Plastics Strategy”, the progress in material design, recycling technologies, or alternative feedstock as a function of three groups of materials, wood polymer composites, polyurethane foams, and rubber recycling products, are discussed. Special attention was focused on polymeric materials and technologies developed in Poland over the last five years. Furthermore, future trends and limitations for “green” polymeric materials are also highlighted. This paper is dedicated for students, researchers, and technologists working (or planning to work) on sustainable development of waste-based polymer materials.

## 2. Wood Polymer Composites

According to ASTM D 883 (“Standard Terminology Relating to Plastics”), thermoplastics are defined as polymeric materials “capable of being repeatedly softened by heating and hardened by cooling through a temperature range characteristic of the plastic, and that in the softened state can be shaped by flow into articles by molding or extrusion for example”. The combination of relatively easy processing and affordable prices of thermoplastics has resulted in their popularity of application as the main matrix, additive, or modifier.

One of the commonly realized trends related to making thermoplastics more sustainable is the production of polymer composites using lignocellulosic fillers [29]—so called “wood polymer composites”. In addition to natural fibers, which are used to increase the strength of the polymer composite [30], the trend of introducing sources of waste lignocellulosic fillers instead of the commonly used wood flour is observed more often. Therefore, in this subchapter, we will focus only on recent advances in this group of thermoplastic materials.

Many papers have shown that replacing a part of the polymer fraction in the composite with a waste filler allows not only to reduce the amount of polymer used, but if it is incorporated into a biodegradable polymer, it does not limit its disposal by biodegradation or composting [30,31].

In recent years, the number of works describing the methods of producing and modifying composites defined as wood-plastic composites (WPC) has continued to increase. Table 3 presents the popularity of polymer matrices used in research that have focused on the development of wood polymer composites. The search keywords were wood polymer composite and WPC in all fields published in 2016–2022, collected from the Scopus^®^ database (data available on 30 January 2022).

The Scopus^®^ database in 2016–2022 showed approximately 1900 records related to wood polymer composites in which the following polymers were used: polyethylene-PE (including bio-PE) (30.1%), polypropylene-PP (19.8%), polylactide-PLA (7.8%), polyvinyl chloride-PVC (5.2%), polystyrene and polyacrylonitrile-butadiene-styrene-PS and ABS (4.0%), polyhydroxyalkanoates and polyhydroxybutyrate PHA and PHB (2.8%), polyamides-PA (1.4%), poly(ethylene terephthalate)-PET (1.3%), polycaprolactone-PCL (1.0%), poly(butylene-adipate-co-terephthalate)-PBAT (0.4%), poly(propylene carbonate)-PPC (0.1%), and others 26.1% (mostly thermosets).

Summarizing the presented data, it can be clearly noted that the most studied and used for WPC production are polyethylene and polypropylene. A gradually increasing share of bio-based grades and PLA from biodegradable polymers also should be noted. These data can be directly related to the availability of these materials and their market share. The share of PP and PE in the global market is 50% [32], while PLA, although it is not a biopolymer produced on the most significant scale (second after PBAT, it is the largest share in production rigid consumer goods [33]. Due to its mechanical properties, comparable to ABS (excluding impact strength) [34], and high availability, PLA is the most frequently used matrix among biodegradable polymers for the production of polymer composites that can compete with PP and rigid PVC-based products.

Most plant fillers in comminuted wooden parts of plants are mainly composed of lignin, hemicellulose, and cellulose. In addition to these ingredients, depending on the type of plant, one can also note starch, proteins, fats and oils, extractive essential oils, and inorganic fractions, including silica [35]. The degradation temperature of the individual components of the lignocellulosic fillers directly translates into the range of the maximum processing temperature and the type of thermoplastic polymer matrix used [36]. The lignin decomposition process occurs in the range of 160–400 °C. At the same time, the degradation of hemicellulose begins at 180 °C and then continues in the range of 200–260 °C with the simultaneous release of significant amounts of gases [37]. Therefore, it has been assumed that the recommended maximum WPC processing temperature is 200 °C and does not exceed 220 °C. For most of the thermoplastic polymers mentioned, the processing temperature usually does not exceed PLA (210 °C), PE (190 °C), and PP (220 °C). The small amount of work on WPC production using polymers such as PA or PET is mainly due to the high melting points of both polymers, i.e., 235 °C and 260 °C, respectively [38].

While initially, introducing natural particle-shaped fillers of plant origin was aimed mainly at making the final products cheaper, while simultaneously accepting a compromise regarding the deterioration of mechanical properties of the final product, some of the published works now focus on the conscious selection of fillers, to obtain the possibility of gaining a more comprehensive modification of the polymeric matrix [39,40,41,42]. It was pointed out that in the case of raw waste lignocellulose-rich fillers from the agricultural and food industry, the impact on the polymer matrix is not only due to the presence of lignocellulosic filler but also from a complex modification resulting from the chemical structure of the filler and low molecular weight compounds that may migrate during the processing as well exploitation of the final composite part.

Among the industries that generate significant amounts of plant-based waste that can be used as fillers for the composite material, one should first mention the agricultural and food industries [43]. The most widely reported materials for producing lignocellulosic waste fillers based on food industry waste are shells, stalks, or other wooden plant parts such as stems [44,45,46]. A considerable amount of research work was also devoted to using fruit and plant waste in the form of seeds, stones, as well as pomace or cake [47,48,49]. It should be noted that both of the aforementioned raw materials are characterized by rather complex material composition, and many research works have been focused on the description of the use of both types of materials as functional fillers, assuming the occurrence of additional modification effects on the polymer matrix. It should be emphasized that also significant amounts of municipal waste in the form of branches or leaves, after appropriate treatment, were used as fillers for thermoplastics [50,51].

### 2.1. Strategies for Compatibilization of Lignocellulose-Rich Filler Reinforced Composites

**Figure 2:**
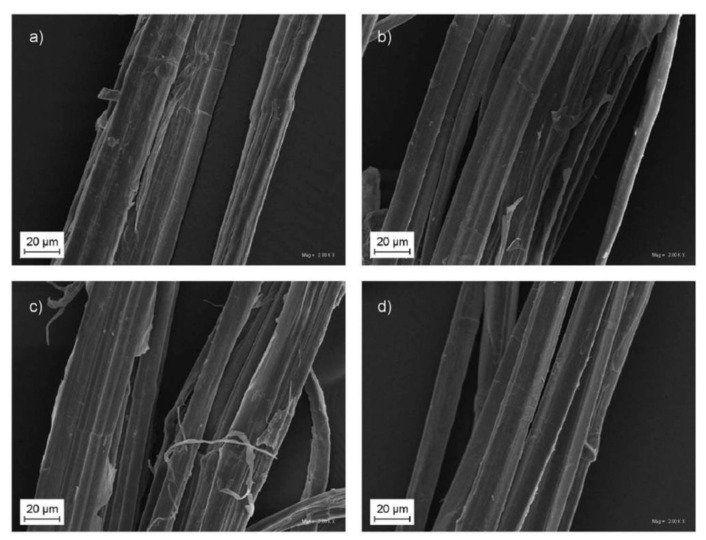
SEM images of hemp fibers: (**a**) untreated; (**b**) silanization; (**c**) acetylation; and (**d**) benzoylation (from [57] with permission from Taylor & Francis).

One of the most critical problems when forming composites with a matrix of thermoplastic polymers is the incompatibility of the filler and matrix, which is usually due to the difference in physico-chemical properties, as the hydrophilic filler is introduced into the hydrophobic polymer matrix [52]. This results in insufficient adhesion at the interface, deteriorating the overall mechanical properties without any beneficial effect, excluding composite stiffening. While the reinforcing effect itself is strongly related to the aspect ratio of the filler [53], the use of an appropriate modification method can significantly reduce the adverse effects resulting from the introduction of raw natural filler to the polymeric matrix. Currently, the most commonly used methods are the production of copolymers grafted with anhydrides, including maleic anhydride grafted polypropylene or polyethylene (PP-*g*-MA or PE-*g*-MA) [54], or the use of fillers surface modification. While in the case of composites modified with vegetable fillers in the form of natural fibers, mercerization and benzoylation methods are commonly used [55,56] to modify the fibers, leading to reducing their specific surface hydrophilicity and changing the degree of crystallinity [57]. Figure 2 shows the microstructure of hemp fibers after treatment by different methods. It can be observed that compared to untreated fiber, the surface of the treated fiber is cleaner, which is due to the removal of waxy substances, lignin, and impurities during treatment procedures.

The effect of silanization, acetylation, benzoylation, and mercerization on the humidity and thermal stability of hemp, flax, and cotton fibers is summarized in Table 4. The results showed that fibers treatment usually reduces their humidity and, at the same time, improves thermal stability, which increases the processing window.

In the case of powder fillers, the more favorable effects are noted when using silanization [58,59], organic acids, and anhydrides [60].

Hejna, Marć, and Korol [61] studied the impact of the type and content of diisocyanate on the structure of modified cellulose fillers. During the research, the most popular isocyanates, isophorone, hexamethylene, toluene, and methylene diphenyl diisocyanate, were used as modifiers. The results confirmed changes in chemical structure and polarity of modified cellulose filler, confirmed by FTIR analysis and stability in different solvents. Moreover, the authors highlighted problems related to volatile organic compounds emission during modifications and storage stability of obtained products.

An interesting approach for further development of filler treatment is based on extrusion or reactive extrusion, which allows continuous modification without solvents and the necessity of further purification. For example, in recent work by Hejna et al. [62], sustainable upcycling of brewers’ spent grain by thermo-mechanical treatment in the twin-screw extruder was investigated. The proposed method allows for, simultaneously, size reduction and change of color of treated cellulosic-rich waste, which can be easily controlled by suitable extrusion parameter adjustment.

Alternatively, for filler surface modification is in-situ reactive processing of polymer and cellulosic fillers, as presented and discussed in works [63,64,65].

However, it should be emphasized that from the point of view of the actual implementation in large-scale production of waste fillers, it is necessary to verify the approach to the production of polymer composites. Adjusting the properties of fillers to polymers makes sense and applies to high-performance composites, where the fillers have circular properties, such as long fibers. However, in the case of composites produced on the basis of waste materials in the form of particles with a low aspect ratio with a low chance of obtaining reinforcing effects, the use of a minimal treatment procedure is justified. Therefore, it is necessary to precisely define additional functional features of fillers that can be used to improve the properties of selected polymers with minimal processing. In order to find a practical and high-tonnage way of utilizing waste from the agriculture and food industry in the process of producing composites, the polymer for the filler should be selected, and not the other way around.

### 2.2. New Functionalities of Natural Fillers in Polymer Composites

In addition to the components of lignocellulosic fillers, including cellulose, lignin, hemicellulose, fat/wax, ash, and water, waste-based fillers from various plant parts can contain many substances that become useful from the point of view of modifying polymeric materials, regardless of the type of used matrix (thermoplastics, thermosets or rubbers). The compounds contained in seeds, shells, stems, skins of nuts, fruits or leaves, fats, essential oils, and flavonoids should be mentioned. Their presence in the compositions and frequent migration to the polymer during the technological process or the final products’ exploitation makes it possible to classify selected waste fillers as functional modifiers/fillers. Among the most effective drug interactions on the polymer matrix reported in the literature in recent years, the ability to act as antioxidants, plasticizers, flame retardants, as well as biocides and antibacterial agents, should be mentioned.

#### 2.2.1. Antioxidant Activity of Waste Fillers

Numerous by-products from plant sources showed antioxidant properties, including coffee silverskin, mandarin and orange peel, hazelnut skin, cocoa, grape and acerola residues, wine seeds, or persimmon peel flour [66,67,68,69]. The work of [70] summarized the possibilities of using by-products from the coffee industry in polymer technology.

The most promising by-product, which does not have a universal solution, is coffee silverskin and coffee grounds [71]. Both products contain compounds with antioxidant activity, including caffeine, epicatechin, and caffeic, caffeoylquinic, feruloylquinic, coumaric, ferulic, gallic, vanillic, and syringic acids. The content of active compounds, which can actively contribute to the greater stability of the composite, is usually below 2 wt.% [72]. The addition of coffee husks, coffee silverskin, or even spent coffee, in the reported cases, showed different tendencies in mechanical properties changes. In the case of polymers such as PLA [73], the addition of coffee by-products as a filler resulted in a depression of the tensile strength, while for PBAT [74] or PPC [75], the presence of the filler resulted in improved mechanical performance even if surface modification of the filler was not applied. While the mechanical properties of the composite in case of addition of coffee silverskin are dependent on the polymer type and interfacial interactions in, according to several studies, additional functionality of increased resistance to oxidation, assessed among the others oxidative induction time, allows it to be classified as a compound with a complex effect on a polymer matrix.

Iyer et al. [76] compare various antioxidant-rich agro-wastes used to increase the thermal stability for low-density polyethylene (LDPE). Grape pomace, orange peel, coffee grounds, and turmeric shavings with the minimalized pre-processing procedure (without any extraction and chemical treatment) showed not only a lack of negative impact on mechanical properties of the composites but also induced significant suppression of chain scission and branching of the polyethylene during multiple reprocessing cycles in a molten state. Despite the necessity of using a higher filler content, i.e., 4–12 wt.%, than in the case of commercial antioxidant (1 wt.% of Irganox I1010), the effectivity on long-term LDPE stabilization was comparable for both groups of the modifiers. The use of waste fillers as antioxidants may reduce adverse degradation effects occurring due to long-term exposure to an elevated temperature in the oxidative atmosphere and also UV light radiation.

Nanni and co-workers [77] showed that PP additive 6 wt.% wine seeds containing significant amounts of polyphenols, including I (þ)—catechin and gallic acid, other simple polyphenols typically present in wine seeds extracts are (-)—epicatechin, (-)—epigallocatechin, (-)—epicatechin gallate, (-)—epigallocatechin gallate (ECG), quercetin, resveratrol, as well as ferulic and ellagic acid, allowing to obtain an UV-protection effect that is comparable to the commercial stabilizer. However, it should be emphasized that despite the comparable effect of raw filler with the polyphenol extract extracted from it, it stabilized PP, and in the case of introducing a natural elongation at break, it was drastically reduced.

Recently, Moraczewski et al. [78] studied the effect of multiple processing of poly(lactic acid) modified by natural anti-aging compounds (coffee, cocoa, and cinnamon extracts). The obtained results were comparable or even better than the results obtained for the synthetic anti-aging compound material, which fully justifies further research and development in this field.

#### 2.2.2. Effects of Oil/Fat-Rich Fillers on the Polymeric Matrix

A significant amount of post-production waste of limited use is generated during edible vegetable oil production from oilseeds. In most cases, they are animal feed additives [79] or, to a small extent, food additives [80,81]. Modified plant oils can serve as functional additives to polymers, including those modifying processing properties (internal lubricants, plasticizers, or slip agents) [82,83] or chain-extenders and compatibilizers, as in the case of epoxidation of linseed or soybean oils [84,85]. Introducing fillers with an increased oil content, which potentially can migrate to the polymeric matrix, was the basis for considering selected by-products as complex modifiers for polymeric composites. Nutshells, stalks, or cakes after mechanical pressing of oil have an oil content of up to 40 wt.%.

Mittal and co-workers [86] decided to use sunflower cake as a filler with a high oil content to produce PLA and PBAT composites. The authors showed the presence of oil in fillers distributed in polymer matrices and its slow migration to the polymer matrix after the molding process during the lifespan, causing changes in their morphology. This was more complex than composites produced only with the lignocellulosic filler. As an agricultural waste with a very high residue of fat/oil that cannot be removed from the lignocellulosic residue by extraction, linseed cake should be mentioned as extremely interesting for polymer modification. A series of works [87,88,89] describes the possibilities of its use as a filler, which, when introduced into polylactide, causes a complex change of material properties. This effect is due to the oil migration from the filler, distributed at various levels in the polymer matrix (macroscopic domains, in the interfacial region, and at macromolecular level), leading to the potentially mutually exclusive phenomena of increasing crystallinity and plasticization of the polymer with a low propensity to form crystalline structures [87]. Interestingly, the oil content in the filler may, on the one hand, increase the compatibility of the composite and its processability by lowering the viscosity compared to defatted grades of the same lignocellulosic filler.

Table 5 presents the effect of linseed cake and linseed cake defatted using acetone extraction on the processing of poly(lactic acid) green composites. As can be observed, in the case of introducing above 10 wt.% of the filler (with a linseed oil content of 28.7%), the significant problems with processability with using melt processing realized on screw-based machines were noted [89]. Therefore, the example of linseed cake shows that application of plant origin by-product may modify both the final and processing properties.

#### 2.2.3. Reduction of the Polymers Flammability

The flammability of lignocellulosic fillers depends mainly on the content of individual components, including hemicellulose and lignin. At the same time, the flammability of natural fiber-reinforced polymer composites with and without the presence of additional flame retardants has been discussed extensively [42,90,91].

Among the two components mentioned, i.e., lignin and hemicellulose, their increased proportion may cause different flame-retardant effects for composites produced with their participation. Lignin is an amorphous component of the wooden parts of plants; it is present in the amount of about 15–30%, containing mainly a polyphenolic structure [92]. It is characterized by high thermal stability and a tendency to create char during thermal degradation. The formation of a temperature-stable charred structure is one of the essential fire retardant methods in polymers modification. Apart from the addition of fillers with increased lignin content, some studies refer to the modification of the separated lignin by introducing additional phosphorus and nitrogen-rich compounds, which proceeded to improve its fire retardancy effect [93].

Wang and co-workers [94] proposed using eucommia residues with 35 wt.% lignin content for manufacturing poly(butylene succinate)-based (PBS) composites. Incorporating 30 wt.% of the filler reduces the peak heat release rate (pHRR) by 46% compared to the unmodified polymer. Moreover, in the case of low filler content (up to 10 wt.%), the total smoke release (TSR) during the cone calorimetry test was lowered by 35%.

Sałasińska et al. [95,96,97] used different approaches to the problem of reducing the flammability of polymer composites containing lignocellulosic fillers based on agriculture and food waste by-products. It related the flame retardancy of the composites filled with nut shells to the formation of expanded char, creating an intumescent effect by hazelnut nut filler that contained high hemicellulose. Contrary to the lignin mentioned above, hemicellulose, particularly the various saccharides it contains, decomposes at low temperatures (from 180 °C) with the simultaneous release of a significant amount of gaseous decomposition products and tar generation. This leads to the formation of a large amount of volatiles, which can be beneficial given the deliberate formation of the cellular structure of the char after exposition to flames. In another work, this effect was amplified by the simultaneous use of 15 wt.% of histidine phosphate complex and the 5 wt.% of grounded hazelnut shell, which allow achieving a developed fire retardant system that is able to form a sizeable swollen structure with numerous closed cells. The modified hazelnut shells compositions reveal similar fire behavior with a simultaneous reduction of TSR compared to pure polymer and composition containing only IFR [96]. The beneficial effect of a plant-derived filler on the reduction of flammability was also described in the case of the addition of raw *Pinus Sibrica* wood for the flammability of epoxy composites [97]. It was shown that the introduction of 20 wt.% wood flour allowed for a 50% reduction in pHRR concerning the unmodified polymer. The release of a significant amount of phenol resulted in a reduction in the flammability of the gaseous mixture during burning; that suggests the complex modification of fire behavior resulting from additional compounds in *Pinus Sibrica* wood.

Another waste filler that showed an additional functionality, mainly reducing the flammability of epoxy-based composites, is spent coffee grounds. Vahabi et al. [98] considered spent coffee grounds and their modification using dimethyl phosphite as a green flame retardant. Results showed that the addition of both pure and modified coffee waste reduces pHRR by approximately 20% and 40%, using pure and phosphite-modified coffee at 30 wt.%, compared to the neo-modified polymer. Moreover, these samples showed the ability to self-extinguish. The differences in the effectiveness of the pure and modified coffee-based filler were related to the additional effect of capturing the free radicals by phosphorous in the gas phase during the effective formation of carbonaceous residue, which suppresses the diffusion of gases and heat to the gas phase.

Hajj et al. [99] compared the properties of flax fabrics grafted by phosphorus flame retardant (vinyl phosphonic acid) using two methods: radiation and chemical treatment. The results showed that thermal stability and flammability of flax fabrics are correlated with the phosphorus content. This research confirmed that radiation grafting is more suitable for low-lignin or lignin-free fibers as flax, cotton, or hemp than chemical treatment. Progress of research in this field was summarized in review work [100].

An interesting approach for modification of cellulosic fillers by flame retardants is an application of ionic liquids [101], which may develop in the near future (considering also using deep eutectic solvents for this purpose).

Another direction for further research in this field is the use of plant compound derivatives to prepare flame retardants with high phosphorous content. One of the most effective solutions is the production of metal phytates. These compounds are produced based on plant-derived phytic acid, a compound commonly found in most cereal grains, legumes, nuts, oilseeds, tubers, pollen, and spores [102]. The results presented in the works [103,104,105] show that metal phytates can be used as a high phosphorous additive to lower the pHRR of modified poly(lactic acid) by up to 30%.

In conclusion, while the addition of organic waste fillers alone can partially reduce the flammability or demonstrate beneficial effects from the point of view of potential flame retardancy, their appropriate modification or use in hybrid systems may allow for obtaining non-flammable polymer composites with increased sustainability, revealing the efficiency of commercial flame retardant systems.

#### 2.2.4. Anti-Bacterial Activity of the Fillers

Plant extracts have been used as antibacterial and agents for a long time, mainly due to the high content of polyphenols [41]. Polyphenolic groups undermine the microbial cell membrane and, afterward, announce cellular elements and cause the cell lysis [106]. The possibility of simplifying the process of obtaining them in order to modify composite materials with a biocidal effect was noticed. Rich in extracts and oils by-products, bioactive compounds such as polyphenols from the food industry began to be carried out to obtain modified polymers that could be used in the food industry for the production of packaging that allows extending the time storage of goods.

El-Nemr and co-workers [106] showed that the introduction of plant-based fillers lignin, separated from sugarcane bagasse and introduced into the PVA/gelatine matrix, showed a significant antibacterial effect against *Escherichia coli*, *Staphylococcus aureus*, *Pseudomonas aeruginosa*, and *Bacillus subtilis*.

Spiridon and co-workers [107] introduced 20 wt.% of the pomace into a polymeric blend of PBAT and corn starch (60/20 wt.%). The addition of waste biomass to the polymer mixture allowed for a significant inhibiting effect of the growth of *Escherichia coli* and *Staphylococcus aureus* bacteria, which in case of the lack of modification, facilitates the growing tendency resulting from a high content of polysaccharides dispersed in the polymeric matrix. This effect was related, among others, to the activity of tartaric acid in pomace, which is known for its antimicrobial effect on *Escherichia coli* [108].

Another work [109] conducted investigations on PLA-based composites with various natural fillers as potential food packing applications. Authors observed that incorporating grape seed in the amount of 3 wt.% into the polymeric matrix reduces the *Escherichia coli* and *Staphylococcus aureus* bacteria activity, with a lack of deterioration of mechanical properties compared to unmodified PLA. It should be emphasized that the low molecular weight compounds contained in the waste lignocellulosic fillers have an anti-bacterial effect. Therefore, it can be concluded that the inhibitory effect of plant-based by-products on the development of bacteria on the surface of composites produced with their participation is a complex effect of the interaction of not only one component migrating from the filler into the matrix but the complex of the filler’s interaction.

## 3. Polyurethane Foams

According to ASTM D 883 (“Standard Terminology Relating to Plastics”), thermosets are defined as “plastics that after having been cured by heat or other means, is substantially infusible and insoluble”. Highly cross-linked structures resulted in the high-performance properties of those materials. This group of polymers includes: phenol-formaldehyde resin [110], urea-formaldehyde [111], polyesters [112], epoxides [113], polyimides [114], and polyurethanes [115].

This review will focus on polyurethanes (PUR), which are synthesized in a polyaddition reaction of multi-functional isocyanates with polyols. PUR materials may be obtained in the form of solid or foamed products with desired properties. PUR is currently the sixth most produced polymer with a global market valued at 55 billion US dollars in 2018 and an annual growth rate estimated at 6.8% for the period 2020–2027 [116]. Due to their structure versatility and properties, PUR materials are used in different forms, such as flexible and rigid foams (more than 60% of the PUR market), coatings, adhesives, and elastomers. Poland is at the European forefront in terms of PUR production. Numerous companies in Poland produce both raw materials for PUR production and various types of PUR materials.

The raw materials for PUR synthesis on an industrial scale come mainly from petrochemical sources. The current strong dependence on non-renewable resources for plastics synthesis is an issue considering the increased CO_2_ concentration in the atmosphere. It is directly associated with the human extraction of fossil resources and it is estimated that by 2050, 20% of the total fossil resources consumed every year will be used in the synthesis of polymeric materials [116]. In addition, fluctuations in crude oil prices cause instability in the price of plastic materials. Both economic and environmental concerns have triggered increasing demand for green technologies, which, in consequence, has led to the use of renewable and waste resources in developing innovative ecological foamed polyurethane materials.

Research on the synthesis of environmentally friendly PUR is carried out in many research centers around the world, including Poland. The development of new PUR materials can be conducted in two ways. The first is to replace petrochemical polyols with bio-polyols synthesized from renewable sources or waste. The second method is the introduction of plant or waste fillers.

Bio-polyols can be obtained from vegetable oils (rapeseed, soybean, palm), microalgae, lignocellulose (wood or annual crops), and polysaccharides. Given the large variety of renewable raw materials, it is possible to obtain bio-polyols with different chemical structures. Lipids are used to synthesize polyester polyols, polysaccharides are employed to prepare polyether polyols, and lignocelluloses find application in the synthesis of aromatic polyols. Arguments are sometimes raised that the use of vegetable oils in the production of bio-polyols is in competition with the production of food. However, it should be taken into account that oil plants can be grown in post-industrial areas where oils cannot be obtained for food purposes. Bio-polyols can also be synthesized from inedible oils such as used cooking oil, tall oil, mustard oil, cashew nut shell liquid, and other sources [117,118,119,120,121].

From the point of view of a circular economy, it is necessary to develop new chemical components based on waste. Used cooking oil is an economic and environmental alternative for fresh oil used in bio-polyol synthesis [122,123,124,125].

Kurańska et al. [126] have evaluated the application potential of used cooking oil in the synthesis of bio-polyols. An analysis of 10 different samples of used cooking oils collected from local restaurants led them to conclude that such waste oils have a similar iodine value, which is the most important property considering the application of waste oil in the epoxidation process. A correlation between the sample type and epoxy value of the epoxidized oil or the hydroxyl value of the bio-polyols was not observed.

Polaczek et al. [127] have analyzed the influence of the palm oil origin on the properties of bio-polyols. Three types of oil samples were analyzed: refined, unrefined, as well as used cooking palm oil. A two-stage method based on epoxidation and oxirane ring-opening was used in the modification of the samples. The bio-polyols were characterized by hydroxyl values from 150–156 mg KOH/g. Another kind of renewable resource for bio-polyol synthesis is biomass [128,129,130,131,132].

Członka et al. [133] have synthesized bio-polyol from walnut shells using the liquefaction process. Starch, cellulose, and lignin are also used to synthesize bio-polyols.

Lubczak et al. [134] have synthesized bio-polyol from starch in an aqueous solution. The products were mixtures of the starch-derived polyetherols and the products of hydroxyalkylation of water. The bio-polyols were used to obtain rigid PUR foams. The authors concluded that the PUR foams had physical properties (apparent density, water uptake, polymerization shrinkage) similar to those of conventional PUR foams.

Szpiłyk et al. [135] have developed a new method of polyol synthesis by cellulose hydroxyalkylation with glycidol and ethylene carbonate. The resultant bio-polyol was used to obtain PUR rigid foams with properties similar to conventional ones, except improved thermal stability of the cellulose-derived foams.

Bio-polyols from cashew nut shells have been successfully synthesized by Ionescu et al. [136] and Gandhi et al. [137]. Abolnis et al. [138] have synthesized bio-polyols from a cellulose production side stream. In their experiment, tall oil fatty acids were obtained through an oxirane ring-opening as well as esterification reactions with different polyfunctional alcohols, such as diethylene glycol, trimethylolpropane, triethanolamine, and diethanolamine. Moreover, in addition to vegetable oils, polyols obtained from the chemical recycling of plastics such as polylactide and PUR are also used for the synthesis of PUR in accordance with the rules of environmental protection [139,140].

A current challenge in chemical technology is also the recycling of waste and the application of such recyclates in the synthesis of new materials. Paciorek-Sadowska et al. [141] have proposed a method of the chemical recycling of PLA waste from 3D printing technology to oligomeric polyhydric alcohols. Glycolysis of PLA waste was carried out at a mass ratio of polylactide to pure glycol of 1:0.5. The resultant eco-polyol was characterized by a hydroxyl number of 326 mg KOH/g, an acid number of 18.12 mg KOH/g, and a water content of 0.331. The chemical structure of the eco-polyol was confirmed by ^1^HNMR, ^13^CNMR, and FTIR. The recyclate was used in a synthesis of rigid polyurethane-polyisocyanurate foams. The partial replacement of the petrochemical polyol with the eco-polyol led to a significant decrease in the apparent density of the foams (from 37.43 to 31.43 kg/m^3^) and compressive strength (from 393 kPa to 303 kPa). For the foams modified with the eco-polyol, a decrease in water absorption (from 6.0 to 1.2%) and brittleness (from 32.1 to 0.6%) was observed.

Borowicz et al. [142] have obtained eco-polyols as a result of a transesterification reaction of PLA waste with diethylene glycol; however, the authors applied a different weight ratio of PLA:diethylene glycol (1:0.4 and 1:0.3). The reduction in the amount of diethylene glycol in the reaction mixture resulted in an increase in the viscosity and a 20% reduction in the hydroxyl value from 262 to 210 mg KOH/g. The eco-polyols were used in a synthesis of polyurethane-polyisocyanurate foams. In those studies, a reduction in the density of the modified materials, as well as a reduction in the brittleness and water absorption were also observed. The authors concluded that the developed method allowed for the fast, cheap, and ecological management of PLA waste and reuse of the final component to synthesize thermal insulating materials.

The use of various methods of modification of renewable raw materials in the synthesis of bio-polyols allows for obtaining bio-components with a wide range of properties, such as hydroxyl number, average molar mass, functionality, and chemical structure. Table 6 presents selected raw materials and waste materials used to synthesize bio-polyols.

A wide range of properties of bio-polyols allows for the modification of both foamed polyurethane materials and solid PUR. The next part of this article presents the latest achievements of mainly Polish researchers in the field of the modification of PUR foams dedicated to thermal insulation applications with renewable and waste materials.

Most rigid PUR foams have a closed-cell structure. However, in recent years, an increase in interest in open-cell rigid and semi-rigid PUR foams has been observed. Polyurethane foams with the open-cell structure are permeable to moisture, have a lower apparent density, and their thermal conductivity is higher (38–40 mW/m·K) in comparison to closed-cell foams (22.0–24.5 mW/m·K) [143].

It should also be highlighted that vegetable and non-edible oils, lignin, itaconic acid, cardanol, gallic acid, eugenol, as well as other waste used for the sustainable development of polyurethane foams are also an ideal alternative to chemical feedstocks in preparing other thermosets polymer materials such as epoxy resins [144,145,146].

Epoxy resins are widely employed for different applications such as construction, coating, automobile, aerospace, and structural adhesives. The current trend for high-performance green materials has resulted in the use of modified natural oils and natural fillers in order to modify their properties.

Sienkiewicz, Czub, and Milo [147] have modified epoxy resins networks with palm oil derivatives. They examined the possibility of using epoxidized palm oil as a modifier of bisphenol A-based epoxy resins. They found that modified vegetable oils (epoxidized or hydroxylated) can successfully partially replace petrochemical resources. Epoxidized oils can replace low and average molecular weight epoxy resins and hydroxylated derivatives of palm oil can substitute for bisphenol A in the conventional method of epoxy resin synthesis. Authors also reported the studies on the rheological behavior of a reacting mixture of modified soybean oil and BPA or low-molecular-weight epoxy resin [148]. Lascano et al. [149] analyzed the kinetic of the curing process of biobased epoxy resin from epoxidized linseed oil by dynamic differential scanning calorimetry. The concluded crosslinking of the epoxidized linseed oil-based epoxy has apparent activation energies similar to other petroleum-derived epoxy resins. The epoxidation process allows one to obtain various epoxidized natural oils that can be used to create a cleaner technology for the production of bio-components, which will be used in modifying thermoset polymeric materials.

The green nature of epoxy resins can also be enhanced by modifying them with fillers [150]. The results of recent research on epoxy resins modified with lignin [151], nanocellulose [152], hemp fibers [153], as well as industrial waste [154] indicated that this trend should develop in the near future.

### 3.1. Open-Cell Bio-Polyurethane Foams

Currently, on the thermal insulation materials market, porous PUR materials obtained from vegetable oil-based bio-polyols, including polyols produced from waste used from cooking materials, are not yet available. Bio-polyols from waste raw materials could become high-quality feedstock in PUR production.

In the literature, the influence of bio-components on open-cell polyurethane foams with a low density (<20 kg/m^3^) has been described by Kurańska et al. [126,143,155].

The reference open-cell PUR foam with an apparent density of 16.3 kg/m^3^ was modified by replacing 20, 40, 60, 80, and 100% of the petrochemical polyol with used cooking oil-based bio-polyols [143]. The content of catalysts and other auxiliary materials was the same in each recipe. The used bio-polyols were characterized by comparable LOH (140 and 159 mg KOH/g); however, their viscosities were different (961 and 3275 mPa·s). The authors explain that such a choice of bio-polyols was motivated by the fact that viscosity is one of the main factors affecting the foaming process. If the viscosity of a component is too high, it may affect the foaming process, which is crucial in the production of porous materials of a very low density. On the other hand, if the viscosity of a PUR composition is too low, the foam may collapse due to the instability of the cellular structure. In foams with an apparent density of about 15 kg/m^3^, the PUR matrix constitutes less than 2% of the volume. The modification of the reference PUR system had a positive impact on both the thermal insulation and mechanical properties of the modified foam materials. The changes were caused by the positive influence of the bio-polyols on the cellular structure. The introduction of even the smallest amount of the bio-polyol (regardless of its type) led to a decrease in cell sizes. The value of the thermal conductivity coefficient for selected foams, including those in which the petrochemical polyols had been replaced entirely with the bio-polyol, was comparable to that offered by commercial materials. A strong influence of the type of bio-polyol on the mechanical properties of the final foams was not observed. The application of the bio-polyols with LOH 140 and 159 mg KOH/g and viscosities of 961 and 3275 mPa·s, positively influenced the properties of the open-cell PUR foams.

Additionally, in order to broaden the application potential of the bio-polyols made from used cooking oil with different characteristics and evaluate their influence on the properties of the open-cell PUR foams, the bio-polyols having LOH 113 (POL100), 198 (POL200), and 254 (POL250) mg KOH/g [155] were also used in the synthesis. The viscosities of those bio-polyols were 670, 4196, and 22,390 mPa·s, respectively. An analysis was conducted of the influence of bio-polyols POL100, POL200, and POL250 on the foaming process of PUR systems dedicated to the production of open-cell PUR foams. In individual recipes, only a selected bio-polyol was used, and, in order to compare the reactivity, the same catalytic system was applied. Based on the analysis of the changes of dielectric polarization, no influence of the bio-polyol type on the reactivity was observed. However, there were substantial differences in terms of the expansion of the PUR systems. In the case of bio-polyol POL100, the foam collapsed, whereas in the system with bio-polyol POL250, it was difficult to pour the material into a form due to the bio-polyol viscosity. A satisfactory structure characterized the material obtained with bio-polyol POL200. A catalytic system was prepared individually for each system to obtain proper samples for further tests. Observations of the performance of the materials confirmed that bio-polyols POL100 and POL250 are not appropriate for the synthesis of open-cell PUR foams. In the case of bio-polyol POL250, the application limitation comes from its high viscosity, whereas in the case of bio-polyol POL100, the problem is related to the viscosity and functionality being too low.

**Figure 3:**
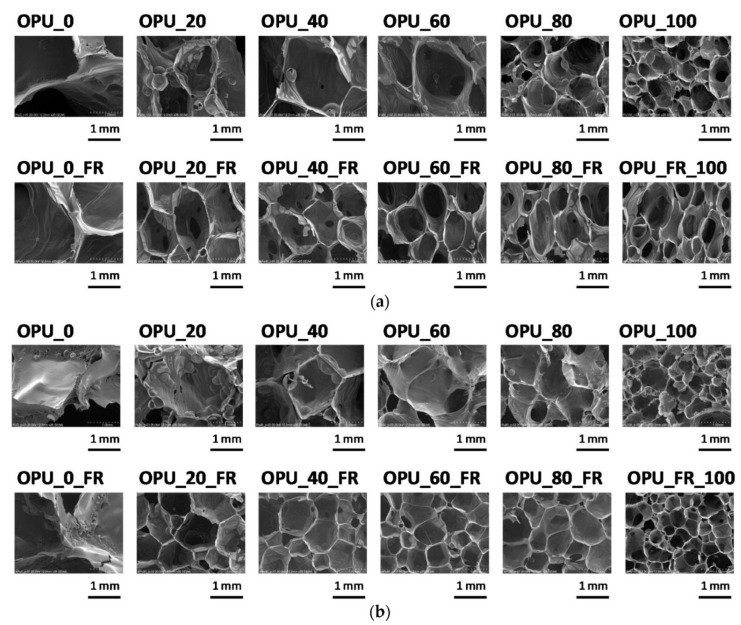
SEM images of polyurethane(PUR) foams modified with bio-polyol: (**a**) Cross-section of the area parallel to foaming direction, (**b**) cross-section of the area perpendicular to foaming direction (Coding: OPU_X_FR, where X is the amount of used bio-polyol, while FR means the use of flame retardant (triethyl phosphate) [156].

In work [156], a negative effect of high viscosity of a bio-polyol was partially mitigated using a flame retardant, which is indispensable in open-cell PUR foams dedicated to construction applications. Tests were carried out in which open-cell PUR foams were modified with bio-polyol having a hydroxyl number of 270 mg KOH/g and a viscosity comparable to that of POL250. Materials in which 20, 40, 60, 80, and 100% of petrochemical polyol were replaced with bio-polyol were produced with and without a flame retardant (triethyl phosphate). Next, selected properties of these materials were found: Apparent density, thermal conductivity coefficient at an average temperature of 0, 10, and 20 °C and mechanical strength at room temperature and −10 °C. What is important is the fact that the influence of temperature on the mechanical strength of the PUR materials was not observed, which is crucial for thermal insulation materials where foams are used in a wide range of temperatures. It was also confirmed that the introduction of an additive flame retardant had a positive impact on the cellular structure of the foams obtained. The microstructure of studied materials is presented in Figure 3.

The results showed that in the case of the foams containing the flame retardant, it was possible to obtain materials with comparable apparent densities, whereas in the materials without this additive, especially those with the highest contents of the bio-polyol, much higher values of this quantity were recorded. That effect was caused by the difficulty of the reaction mixture to expand due to the high viscosity of the PUR system without the flame retardant. Based on the studies of the influence of bio-polyols of different chemical structures obtained through epoxidation and oxirane ring-opening on the properties of open-cell polyurethane foams, it concluded that the most favorable is the use of bio-polyols having LOH values of 150–200 mg KOH/g and viscosities in the range 950–4200 mPa·s.

As mentioned before, thermal insulation materials for applications in construction must be characterized by reduced flammability. To this end, the materials containing 100% of bio-polyol UCO_TEA and UCO_DEG were modified with three types of flame retardants: (tris(1-chloro-2-propyl)phosphate), (TCPP), dimethyl propylphosphonate (DMPP), and triethyl phosphate (TEP). The flame retardants differed in terms of the phosphorus content, and one had chlorine in its structure [157]. For over 10 years, at numerous scientific and technical conferences, the idea to ban TCPP due to the presence of chlorine has been discussed. However, most of the commercial PUR systems contain this effective flame retardant. Another advantageous flame retardant is DMPP, given its high content of phosphorus. PUR systems were modified by introducing 10, 20, and 30% of flame retardant with respect to the polyol mass. The influence was analyzed of the flame retardants on the reactivity of the system, apparent density, cellular structure, thermal conductivity coefficient, compressive strength, flammability, and thermal stability; in addition, volatile products during thermal degradation were identified.

The introduction of flame retardants led to a slight decrease in the reactivity of the PUR systems. In the case of TCPP, the reactivity changes were the smallest. It was observed that flame retardant TEP increased the content of closed cells in the system containing bio-polyol UCO_TEA. In the case of that system, during industrial tests, it was necessary to make a correction in the recipe in terms of the surfactant content. The unexpected effect of the increased content of closed cells highlights the need for a comprehensive analysis of the influence of each additive on the properties of open-cell PUR foams. The most advantageous values of the thermal conductivity coefficient were obtained in the case of the materials modified with flame retardant DMPP, which is connected with favorable cellular structures of the foams modified with this flame retardant. As far as flammability is concerned, the best reduction effect was also observed in the case of DMPP. Nevertheless, the other materials are also characterized by an oxygen index higher than 21%, which means that the materials do not sustain the burning process in the air after the flame is removed. Moreover, open-cell PUR foams containing bio-polyol UCO_TEA exhibited lower flammability due to the presence of nitrogen atoms in the structure of this bio-polyol.

### 3.2. Closed-Cell Bio-Polyurethane Foams

Rigid PUR foams with a closed-cell structure are mainly used as insulation materials owing to their low thermal conductivity coefficient (lower than that of foamed polystyrene or mineral wool), high dimensional stability, good mechanical properties, and low apparent density [158]. Partial replacement of petrochemical polyol with bio-polyols from waste or renewable resources has become an increasingly popular approach over the last decade. The method and conditions of the synthesis of bio-polyols have an influence on the foaming process of PUR systems and the properties of rigid foams. The most important properties are the hydroxyl value, viscosity, molecular weight, functionality, and position of hydroxyl groups in the triglyceride chain.

The bio-polyols obtained from used cooking oils, both through the method of the epoxidation and oxirane ring-opening as well as transesterification, were tested in systems dedicated to the production of closed-cell PUR foams [158,159]. The formula of the reference system was modified by replacing the petrochemical polyol with bio-polyol (having LOH of 295 mg KOH/g obtained by epoxidation and oxirane ring-opening) in an amount of 20, 60, and 100% [158]. The influence of the bio-polyol on the foaming process of the PUR system was analyzed along with the performance properties, such as thermal conductivity coefficient, mechanical strength, dimensional stability, and thermal stability.

Based on these studies, it was concluded that the bio-polyol synthesized from used cooking oil does not affect the reactivity of the PUR system. This effect is different from what has been described in the literature so far. The cause may lie in the high viscosity of the bio-polyol. It was found that in the case of the closed-cell foams, a complete replacement of the petrochemical polyol is not possible due to the tendency of cells to open, which has a negative impact on the thermal conductivity coefficient and the mechanical properties of the modified foams. The same conclusion was reached in the case of the PUR foams modified with bio-polyols obtained in the transesterification reaction of used cooking oil with diethylene glycol and triethanolamine [159].

### 3.3. Polyurethane Composite Foams

In order to improve the mechanical properties or decrease the flammability of porous PUR materials, they are modified with organic and inorganic fillers such as nanoclay, expandable graphite, basalt fiber, talc, or polyhedral oligosilsesquioxanes (POSS) [160]. Vegetable fillers and waste fillers are also introduced in order to reduce the consumption of raw materials for the production of polyurethanes, which in turn may reduce the price of the finished product.

Microfillers can act as nucleating agents during the foaming process. It was confirmed in the work of Uram et al. [161], where the authors modified rigid PUR bio-foams with microcellulose. PUR foams containing 40 wt.% of rapeseed oil-based polyols were modified with the natural filler in amounts of 1, 2, and 3 php (per hundred polyols). It was found that the addition of the cellulose filler increased the number of cells in the foams.

Following the idea of a circular economy, it is important to use waste fillers. Many scientists have carried out research on the use of fallout fillers to modify rigid polyurethane foams.

Członka et al. modified rigid PUR foams with buffing dust [162], a proteinous solid tannery waste, and potato protein [163], the main product of thermal and acidic coagulation of potato juice obtained from potatoes during starch production. Buffing dust was added in an amount of 0.1–5 wt.% in relation to the total polyol mass. The authors noticed that the PUR foams modified with 0.1 wt.% of buffing dust exhibited favorable properties. The higher contents of the filler caused a wider cell size range and higher cell size distribution compared to the unmodified foam. In the case of potato protein, the addition of 0.1 wt.% of potato protein improved the compressive strength and had a beneficial effect on the thermal conductivity and water absorption. The cited papers confirm that the addition of fillers over a certain optimal level leads to deterioration of the physico-mechanical properties due to foam morphology.

In the case of natural fillers, their main disadvantage is their hydrophilic nature that limits their compatibility with the hydrophobic polyurethane matrix. This can lead to increased water absorption, which limits their use as a thermal insulation material. Additionally, on the surface of the natural fillers, there are wax substances that block reactive functional groups. Various modifications are made to improve adhesion to the polyurethane matrix [164]. Poor interphase interaction between the PUR matrix and filler surface makes cells collapse under the pressure of the neighboring cells leading to the formation of open cells [165].

Członka and Strąkowska [166] modified rigid polyurethane foams with 2 wt.% of non-treated, acetylated, and silanized walnut shells (WS). The morphology analysis confirmed that the addition of WS promoted a reduction in cell size compared to unmodified PUR foams. The authors observed the most significant improvement of mechanical properties in the materials modified with silanized WS. The compressive, flexural, and impact strength were enhanced by 21, 16, and 13%, respectively. The addition of fillers did not affect the insulating properties of modified foams.

In other studies [167], rigid PUR foams were modified with 2 wt.% hemp shives (HS) fillers. Three types of HS fillers were tested–non-treated HS, HS impregnated with sunflower oil (SO), and HS impregnated with tung oil (TO). Depending on the properties tested, it was observed that the unmodified filler had a positive effect on the mechanical properties, while the incorporation of impregnated HS fillers resulted in an improvement of thermal stability and flame retardancy. The rigid PUR foams containing impregnated fillers were characterized by decreased the value of heat peak release (pHRR), total smoke release (TSR), and limiting oxygen index (LOI).

Recently, Hejna et al. [168,169] studied the effect of treated ground tire rubber (GTR) (treatment by various natural oils, KMnO_4_, or H_2_O_2_) on polyurethane/GTR composite foams. Presented results showed that suitable modification of ground tire rubber is a promising approach for tailoring the structure and performance of polyurethane composite foams.

Table 7 presents the filler selected for the synthesis of bio-composites and the most important conclusions of their research work.

### 3.4. Polyurethane Recycling

The rising amount of polyurethane foam waste in the environment has enforced growing research attention onto circular economy solutions to closing the material loop. The main goal of chemical recycling is to recover polyol as the main product by breaking down the urethane bonds under controlled reaction conditions. In this case, high molecular, cross-linked polyurethane is chemically broken down into lower molecular weight liquid products, which can be used during the manufacturing of polymeric materials, e.g., polyurethanes or epoxides [175]. Chemical recycling is the most sustainable recycling method due to its economic and environmental advantage compared to landfilling or burning.

Considering environmental protection, the possibility of recycling polymeric materials should be taken into account in the design stage. The current state of knowledge on the recycling of thermal insulation materials includes the chemolysis of polyurethane materials derived from petrochemical feedstocks.

It is known that the chemolysis processes, such as glycolysis, hydrolysis, and aminolysis, can be used for depolymerization and recycling of polyurethane [176,177,178]. Moreover, the glycerolysis of polyurethane wastes is also considered as a promising method of waste polyurethane recycling [179,180].

Major differences between glycolysis, glycerolysis, hydrolysis, and aminolysis are in the type of reactants utilized for depolymerization and the composition of final products. In the case of hydrolysis, water is utilized for decrosslinking the polymer, requiring a relatively high reaction pressure and temperature. The hydrolysis of polyurethanes is usually carried out at temperatures higher than 200 °C and pressures higher than 16 bars. The hydrolysis products are original polyols and amines (isocyanate derivatives). Glycolysis or glycerolysis can be carried out at atmospheric pressure at about 200 °C (between 180 and 240 °C). Obtained products are original polyols, isocyanate-containing polyols, and residual glycolytic agents (reactants). Aminolysis can be carried out at atmospheric pressure and temperature much lower than that required for glycolysis. Aminolysis products are disubstituted ureas and original polyols. Most of the chemolysis products are described to be used in polyurethanes without any modification or with a minimum modification. Practically all chemolysis processes, including glycolysis, result in amine formation, which greatly affects the reactivity of the chemolysis products with isocyanates.

The most important chemolysis process is glycolysis because it provides the best outcomes with respect to the quality of the recovered product at mild reaction conditions. The glycolysis of polyurethane foam is the most environmentally friendly and widely-utilized process reported in the literature with the highest development in terms of research and technological aspects. Glycolysis of polyurethane foam consists of a transesterification reaction. If the structure of polyurethane is conceptually simplified, the glycolysis mechanism can be described as an intermolecular exchange in the urethane group through an addition-elimination process, where hydroxyl end groups of the glycol act as a nucleophilic agent. Glycolysis processes have been described for a great variety of polyurethane products, including rigid and flexible polyurethane foams.

Glycolysis is a process of polyurethane degradation leading to a breakup of urethane and/or urea groups in specific conditions under the influence of glycols or mixtures with amines. Low molecular weight glycols with of up to six carbon atoms (ethylene, propylene, butylene, diethylene, and dipropylene glycols) as well as polyethylene and polypropylene glycols are used the most frequently [181,182]. The type of compounds used in glycolysis has a decisive influence on the time and temperature of the reaction, the tendency of the reaction mixture to separate into independent phases, and the compositions of those phases. The products of the reaction are various compounds that end with hydroxyl and/or amine groups.

The reaction conditions influence the quality of the product, which is why many researchers have investigated the mechanism, the effect of glycol, type, and concentration of catalyst, as well as temperature [183,184]. Another important factor is the chemical structure and the molar mass of the polyol used in the synthesis of polyurethane foams.

Currently, the polyols obtained from split-phase glycolysis show better properties and have a higher purity compared to the polyols recovered from single-phase glycolysis. Split-phase glycolysis is mainly obtained by using a stoichiometric excess of glycols for the recycling of polyurethanes containing high molecular weight polyols. The solubility of the polyols in the glycol plays an important role in the separation of two phases. By this method, the upper phase consists mostly of the recovered polyols and the bottom phase is the excess glycol and other products of the reaction [185].

Del Amo et al. [186] analyzed the influence of transesterification agents (diethylene glycol and crude glycerol) on the chemolysis process of rigid polyurethane foams. The authors concluded that diethylene glycol did not allow for achieving a split-phase process, obtaining a product with low purity (61.7 wt.%). On the contrary, crude glycerol allowed for achieving split-phase glycolysis, improving the recovered polyol purity (76.5%). A similar comparison of the effects of the transesterification factors but in the case of elastic foams was made by Simon et al. [187]. As a result of the chemolysis reactions, two phases were obtained. The upper phase was mainly constituted by base polyol, whereas the bottom phases consisted of several glycolysis byproducts and the excess of the transesterification agents. Crude glycerol provided an upper phase with a lower concentration of by-products and glycolysis agent and, as a result, with a higher proportion of the recovered polyol, which has a similar hydroxyl number than the raw polyols without the need of purification. Therefore, the use of glycerol increases the polyol recovery yield in comparison with the performance shown by the diethylene glycol. This is related to the higher dielectric constant of the glycerol.

In another work, Simon et al. [188] confirmed that the chemical structure of the petrochemical polyols used in the synthesis of foams that were next used in chemolysis has an influence on the properties of the recyclates. In the synthesis of elastic foams, they used polyether polyol and polymeric polyol. After the glycolysis reaction was carried out under the same conditions for both materials, they concluded that the upper layer of the recyclate obtained from the polymeric polyol-based foam was characterized by a hydroxyl number of 200 mg KOH/g, whereas in the case of the polyether polyol-based foam, it was 352 mg KOH/g. It can hence be expected that bio-polyols of various chemical structures and properties dedicated to synthesis will lead to the production of recyclates of different characteristics.

The reagents used in the chemolysis process of elastic foams, such as EG, DEG, MPG PEG, and glycerin, are also used in the case of rigid polyurethane foams. The literature data indicated that the most popular catalysts dedicated to the chemolysis of polyurethane foams are DEA, KAc, NaOH, Sn(Oct)_2_, Zn(Ac)_2_, and triethanolamine.

The main products of polyurethane glycolysis are compounds with a structure similar to that of the initial polyol, containing urethane bonds, low molecular weight mono- and dicarbamates, amines, and urea oligomers. In the case of polyester-urethanes, during glycolysis there is an additional transesterification reaction of ester bonds in elastic segments and as a consequence, the molar mass of the obtained product decreases.

In the literature, there is a lack of comprehensive papers related to the process of the chemical recycling of polyurethane foams derived from vegetable polyols, which shows the necessity to find the optimized processing conditions, suitable catalysts, and diol-to-waste ratios.

## 4. Rubber Recycling

According to ASTM D 883 (“Standard Terminology Relating to Plastics”), an elastomer is defined as “a macromolecular material that at room temperature returns rapidly to approximately its initial dimensions shape after substantial deformation by a weak stress and release of the stress.” For comparison, rubber is defined as “a material that is capable of recovering from large deformations quickly and forcibly and can be, or already is, modified to a state in which it is essentially insoluble (but can swell) in boiling solvents, such as benzene, methylethylketone, and ethanol-toluene azeotrope”.

Nowadays, sustainable development of this group of polymeric materials can be divided into three main directions: (i) elastomers with unique properties dedicated for special applications [189,190,191,192,193]; (ii) green additives in rubber chemistry and technology [194,195,196,197,198]; and (iii) waste rubber recycling [199,200,201,202,203].

Estimated data indicates that general rubber goods (e.g., belting, hoses, tubes) and consumer products (e.g., footwear, toys, sport, and leisure equipment) are around 30% of the rubber marker. However, only 1.5% of rubber materials from this sector are recycled or reused [204]. So far, several attempts have been made to resolve the technological problems related to shoes [205,206,207] or gloves recycling [208,209,210]. It seems that there is an increasing demand of industry for the application of circular economy strategies that should enforce the development of technologies based on this stream of waste rubbers (general rubber and consumer products) in the near future.

At present, the main source of waste rubbers generated to the environment is waste tires. Estimations showed that at present, each year, around 1000 million tires are not suitable for further use or retreading [211]. Moreover, until 2030, the number of waste tires generated to the environment will increase to 1200 million tires/per year [212]. If the estimated data are correct and the development of rubber recycling technology remains relatively low, an increase of 20% of waste tires within the next 10 years will be observed.

Recently, Przydatek, Budzik, and Janik [213] showed that during the last 11 years, over 5 million tons of waste tires were generated in Poland, exceeding the number required for 50 million registered vehicles. Presented statistical data showed that the tire accumulation indicator on a national level is 2.12 tons/km^2^, while in a smaller voivodeship value of this parameter is almost 24 times higher (48.06 tons/km^2^). This indicates a necessity for increasing the points responsible for the collection of waste tires, as well as their further management and recycling.

Therefore, this subchapter focused on recent advances in green technologies in rubber recycling and sustainable development of advanced materials with ground tire rubber (GTR) as a promising approach for waste rubber upcycling.

### 4.1. Green Technologies in Rubber Recycling

#### 4.1.1. Grinding and Pulverization Technologies

According to recent statistical data published by European Tyre and Rubber Manufacturers’ Association (ETRMA), in 2019, around 74.1% of waste tires generated in the European Union, United Kingdom, Norway, Serbia, Switzerland, and Turkey were recycled by granulation [214]. This indicates that these grinding and pulverization technologies are the most common and basic methods of rubber recycling. Products of resulted from waste tires disintegration can be divided into three groups: steel, textile cord, and ground tire rubber (GTR). Depending on the grinding or pulverization technology and used grinding conditions (e.g., shear forces, temperature), GTR particles are characterized by different particle size distribution, average particle size, surface characteristics, etc. [24,203].

Moreover, the final performance properties of GTR are also affected by suitable segregation of tires, which are usually classified as passenger car tires and truck tires (sometimes in the literature also off-road tires are included in this classification). In comparison to passenger car tires composition, truck tires are made with a higher content of natural rubber and a lower level of carbon black, which is related to obvious differences in performance of those two types of tires. Moreover, it should be highlighted that natural rubber is more prone to thermal degradation than synthetic rubbers [215,216], and as a consequence, its degradation or partial devulcanization/reclaiming might occur during the grinding process.

Tyromer Inc. [217] indicted that the tensile strength of reclaimed rubber obtained from the tread of truck tires is around 33–83% higher than reclaimed rubber prepared from passenger car tires. In addition, other sources indicated that the chemical composition of rubber waste affects (more or less) the final processing and performance of matrices, e.g., bitumen [218,219,220], polyethylene [221], or natural rubber [222]. The above-mentioned information confirmed that suitable segregation of waste tires before grinding is crucial for the repeatability of the process and good quality of obtained products [223].

At present, the most popular methods of waste tires grinding are performed at ambient and cryogenic temperatures. It should be mentioned that during waste tires grinding at ambient temperature, processed material can reach up to 130 °C [224]. Therefore, additional cooling by water is usually necessary. GTR obtained by this method is characterized by an irregular particle shape as well as a spongy and well-developed surface. On the other hand, waste tires grinding by the cryogenic method is performed below their glass transition temperature. In these conditions, the elastic rubber material is converted into a frozen brittle form and subsequently crushed using a hammer mill. GTR obtained by cryogenic methods is regular, smooth, and has a low surface area [225].

Due to usually limited access to liquid nitrogen, its costs and high demands regarding storage and ambient temperature methods are more popular. According to our knowledge, all industrial lines dedicated to recycling waste tires in Poland are based on ambient temperature methods. Commercially available GTR characteristics are usually based only on the particle size distribution and average particles size as the main technological parameters defined by producers. The smallest particle size available in Poland is currently below 0.6 mm (GTR obtained via ambient grinding method). For example, Grupa Recykl SA from Poland offers products with the tradename Green Gran with a variable particle size distribution, from 4–7 mm to less than 0.6 mm (Green Powder). Table 8 shows the example of particle sizes available for ground tire rubber produced by Grupa Recykl SA.

Seghar et al. [227] provided interesting information about the estimated costs of waste rubber grinding and showed that for waste rubber particles 1–3 mm, the cost is around 120 EUR/ton, for a size 0.8–2.5 mm, it is 130 EUR/ton, while for waste rubber particles lower than 0.8 mm, the price is 300 EUR/ton. The authors suggested that for the devulcanization of waste rubber, a reasonable economic size is 0.8–4 mm.

Isayev, Liang, and Lewis [228] studied the effect of the GTR particle size on the efficiency of ultrasound-assisted thermo-mechanical devulcanization of GTR using a co-rotating twin-screw extruder. The results showed that using GTR with an average particle size of 0.595 mm resulted in a lower die pressure and higher devulcanization level compared to the trials with GTR-2.0 mm. The authors indicated that a higher devulcanization level improves processing obtained products; however, at the same time, it had adversely affected the modulus and tensile strength of revulcanizates. Similar observations were described in works [229,230]. Research work of de Sousa et al. [231,232,233] showed that the level of the devulcanization/degradation of ground rubber (GTR, natural rubber) might affect reversion during vulcanization and, consequently, the mechanical properties of vulcanizates.

Moreover, it should be highlighted that the devulcanization level of waste rubber also has a significant impact on matrix/GTR interfacial interactions and, as a consequence, the processing and performance properties of prepared materials [234,235,236]. Therefore, the devulcanization level should also be considered during the manufacturing of polymer blends or composites modified by GTR.

Recently, the development of different waste rubber grinding or pulverization methods is gaining increasing attention. For example, Kroll and Hoyer [237] presented a device called “Reaktruder” with a construction similar to an extruder dedicated to the grinding of elastomers by small- and medium-sized companies. Estimated calculations showed that applications of this technology pay off after approximately 1500 h of device operation and can be an auspicious approach for recycling high-cost elastomers (e.g., fluorocarbon rubber, ethylene-acrylate rubber).

Dobrotă and Dobrotă [238] showed that the grinding of waste rubbers in the presence of ultrasounds reduces energy consumption and increases the production efficiency. Moreover, the results showed that obtained ground rubber (average particle sizes in the range of 100–150 μm) could be successfully used to replace reclaimed rubber during the manufacturing of new rubber compounds.

Holka and Wełnowski [239] constructed the whole station for shredding and granulation of whole tires. The grinding device is based on special discs under the trade name Rotarex. The proposed solution takes up a small space (the size of a container), allowing easy transport (e.g., collection points for waste tires or illegal dumping) without complicated logistic operations.

Wang et al. [240,241] investigated the quality of GTR (e.g., particle size distribution; average particle size; cross-link density) and sol fraction as a function of water-jet pulverization parameters. The results showed that the average particle size of GTR obtained by water-jet pulverization was below 200 μm, while the microstructure of GTR indicates that high compressive shear forces acting on cross-linked rubber during water-jet treatment might result in partial reclaiming on the GTR surface.

In work [242], Holka and Jarzyna presented the summary of their research on applying water-jet technologies in waste tires recycling. The main finding showed that adjusting suitable parameters allows the reduction of energy consumption of water-jet disintegration of waste tires compared to conventional mechanical methods (the difference is ~58%). Moreover, the authors pointed out that products prepared by water-jet technology are characterized by a lower content of impurities than those prepared by mechanical methods.

Bowles et al. [243] made the comparison between characteristics of GTR obtained by an ambient method with GTR obtained by ultra-high-pressure water-jet pulverization. The results confirmed that natural rubber/styrene-butadiene rubber-based composites modified with GTR obtained ultra-high-pressure water-jet pulverization had higher performance properties than those with GTR obtained via ambient grinding. However, it should be mentioned that the improvement of tensile strength or tear strength in comparison to the reference sample (without GTR) was not observed by the authors.

In work [244], Zefeng et al. showed that using GTR obtained by ultra-high pressure water-jet pulverization (with an average particle size of 270 μm) had a negative impact on the processing of natural rubber-based composites. It was found that tensile parameters improved after incorporating GTR up to 40 phr for study materials, which was related to good dispersion and compatibility between GTR and natural rubber.

Park et al. [245] compared the GTR microstructure prepared for shear grinding and cutting grinding. The results showed that GTR particles obtained via shear grinding were characterized by a higher surface area than GTR obtained via cutting grinding, which affects the tensile strength of reactively sintered GTR (particle size in the range: 1–2 mm). The authors demonstrated that GTR via shearing showed a tensile strength of 3.2 MPa, while for GTR via the cutting value of this parameter was 2.5 MPa. The grinding method has a negligible impact on the hardness of studied materials (~63 Shore A).

Recently, Hrdlička et al. [246] presented research about rubber compounds modified with variable fractions of GTR produced via three methods: ambient, cryogenic, and water-jet milling. The best performance properties for rubber compounds modified with GTR (tensile strength: 18.4 MPa and elongation at break 459%) were achieved using particles prepared by water-jet milling with a particle size in the range of 0–100 µm. The authors indicated that waterjet milling of waste tires in fine particles with a high specific surface disperses well into the rubber matrix.

It seems that waste rubber grinding should develop in the near future, especially those based on continuous and high-effective methods (e.g., extrusion), which allow for additional devulcanization and/or modification/functionalization of GTR during grinding or pulverization, e.g., by using supercritical carbon dioxide [247,248].

#### 4.1.2. Low-Temperature Devulcanization and Modification of GTR

Recent trends showed that low-temperature devulcanization and combination of devulcanization with suitable modification or functionalization are gaining more attention. However, regardless of the used method, the obtained products (reclaimed/devulcanized rubber) should fulfill at least two requirements: (i) flowability and (ii) ability to be vulcanized (revulcanized) [249,250].

Due to huge progress in the low-temperature devulcanization technologies over the last five years, in this subchapter, we present only selected examples to highlight the possibilities for further development in this field.

The main advantages of the low-devulcanization temperature of waste rubbers are: (i) Reduction of energy consumption necessary for the disintegration of three-dimensional network present in waste rubbers; (ii) better control of process selectivity and limited main chain degradation; and (iii) limited volatile organic compounds emission during the waste rubbers treatment [251,252].

Shi et al. [253] studied structure-property relationships of reclaimed rubber obtained by different methods: Low-temperature shear reclaiming and high temperature reclaiming via two-roll milling; reclaiming via twin-screw extrusion and reclaiming in the presence of supercritical carbon dioxide. The comparison of used methods indicated that the optimal conditions for GTR reclaiming are relatively at a low temperature, rather at low shear forces, and an oxygen-free atmosphere.

**Figure 4:**
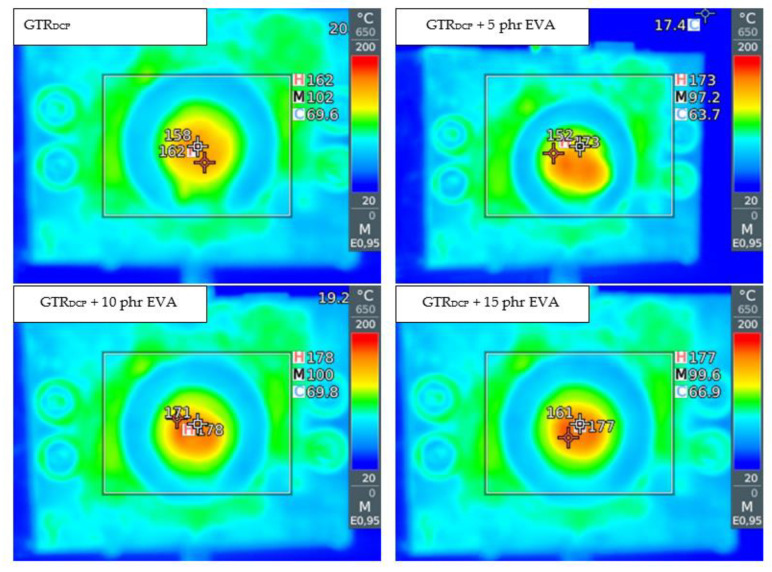
The infrared camera images for modified ground tire rubber (GTR) samples: GTR_DCP_; GTR_DCP_ + 5 phr EVA; GTR_DCP_ + 10 phr EVA; GTR_DCP_ + 15 phr EVA (DCP—dicumyl peroxide, EVA copolymer with 18% vinyl acetate) (adopted from [255]).

Saiwari, Dierkes, and Noordermeer [254] performed a comparative investigation of the devulcanization parameters of rubbers used in the tire industry (natural rubber, styrene-butadiene rubber, butadiene rubber, and chlorinated butyl rubber). The main findings showed that the main processing parameter affecting rubber devulcanization mechanism and efficiency is temperature. The authors indicated that it is necessary to keep the devulcanization temperature as low as possible for the efficient devulcanization of tire rubbers. Figure 4 presents the infrared camera images for modified GTR, which confirms that the kind of used modifier might have a significant impact on the temperature of materials, especially when the process is performed in auto-thermal conditions [255].

To fully understand the effect of temperature on devulcanization efficiency, two aspects should be considered: The temperature of the material during processing and efficient cooling of reclaimed rubber. However, detailed information on the last aspect is usually omitted in the literature.

The main drawbacks of low-temperature devulcanization are technological aspects related to the processing of cross-linked rubber [256]. One of the solutions to this issue is a modification of GTR using plasticizers [257,258] or thermoplastics [259,260].

Zedler et al. [261] presented comprehensive research about the role of polyoctenamer rubber (additive commonly used in rubber recycling) and curing additives on the processing and performance properties of modified GTR. The results showed a deterioration of physico-mechanical properties of the GTR/polyoctenamer rubber system compared to pure GTR, which is due to a lack of cross-linking between GTR and polyoctenamer rubber. Moreover, it was found that the presence of polyoctenamer rubber without a curing system hinders the sintering process between GTR particles. The highest tensile strength (~5.5 MPa) was determined for a GTR/polyoctenamer rubber system cured with sulfur.

GTR modification by microwave treatment is a promising approach for further development of waste tires recycling technologies [262]. Microwaves radiation provides a precise amount of energy in a specific location. This allows more efficient control of chemical reactions, which results in improved productivity and economic competitiveness of microwave treatment compared to other conventional heating methods.

Paulo et al. [263] studied the microwave treatment of GTR with inorganic salts (CuSO_4_, ZnCl_2_, CdCl_2_, and Bi(NO_3_)_3_) and nitric acid. It was found that using metallic ions and nitric acid enhanced the microwave devulcanization and partial oxidation of GTR (the exception was CdCl_2_).

Seghar et al. [264] investigated the effect of the ionic liquid (pyrrolidinium hydrogen sulfate) on microwave treatment efficiency of styrene-butadiene rubber vulcanizates. It was found that ionic liquid improves microwave treatment of waste rubber, which resulted in the higher temperature of material exposed to microwave treatment. The authors pointed out that promising approaches for further development are methods combined with the mechanical and microwave treatment of waste rubber.

**Figure 5:**
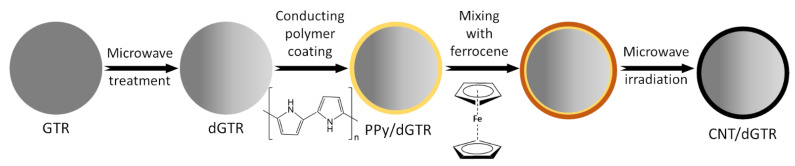
Procedures for synthesis of devulcanized GTR modified with carbon nanotubes (CNT) (CNT/dGTR) (Redesigned based on [265]).

Poyraz et al. [265] proposed the protocol of modification of GTR by carbon nanotubes (CNT), which is presented in Figure 5. In the first step, GTR is treated by a microwave treatment to provide its partial devulcanization and increased mobility of rubber chains into the GTR surface. In the second step, devulcanized GTR is coated with conducting polymer (polypyrrole) and mixed with organic metallocene precursor (ferrocene). Subsequently, coated GTR is treated by microwaves, which results in the in-situ formulation of CNT on the GTR surface.

Jia et al. [266] indicated that for GTR/CNT composites, the typical segregated structure with CNTs nanoparticles is selectively localized at the boundaries of GTR domains. The authors showed that GTR modified with only 5 wt.% CNT exhibits a high electrical conductivity of 109.3 S/m and huge potential to be applied as low-cost, flexible electronic devices.

Liu et al. [267] used a variable content of barium ferrite ultrafine powder (in the range of 0–50 wt.%) for the modification of GTR via microwave treatment. The results showed that increasing the content of barium ferrite improved microwave devulcanization and activation of GTR, which resulted in better compatibility between GTR and the used filler. The prepared modified GTR can be applied as a low-cost, flexible magnetic material, promising for upcycling waste tire rubber.

An interesting alternative for microwave treatment of waste tire rubber is surface treatment by plasma. However, the number of papers published is rather limited [268,269,270].

Many papers have focused on GTR modifications based on batch and multi-step protocols with a necessity of using solvents or purification of obtained products [271,272,273]. Another problem in GTR modifications is usually the long-time necessity to perform suitable changes in the GTR structure, which has caused problems with their further application at an industrial scale. Therefore, the batch methods can be useful for preliminary treatment of waste rubber before extrusion, two-roll milling, or high-shear speed mixing.

At present, the most promising approach for further devulcanization and suitable modification/functionalization of GTR are methods based on extrusion or reactive. This technique allows the preparation of reclaimed rubber [274] or novel products such as reactive plasticizers [275], thermoplastic elastomers [276], or bitumen modifiers [277], which, after upscaling and industrial trials, can be produced with higher throughput. Therefore, further research on reactive extrusion of waste tire rubber is fully justified and should develop in the near future.

### 4.2. Sustainable Development of Advanced Materials with GTR

#### 4.2.1. Self-Healing, Shape-Memory, and Recyclable Materials

Self-healing, shape-memory, and recyclable materials modified or based on waste tire rubber is a relatively new field of research. The number of published data is somewhat limited. However, considering current trends in the development of advanced elastomers [278,279,280], it seems that applying GTR into material or composites with special or unique properties is a promising approach for waste tire upcycling, which should be explored in the near future.

Hernández Santana et al. [281,282,283] investigated the possibility of combining self-healing properties using GTR. The main findings indicate that for styrene-butadiene rubber/GTR systems, a semi-efficient sulfur curing system gives the best balance between tensile strength and healing. Moreover, applying a coupling agent improves mechanical performance without negligible impact on healing. Healing efficiency was between ~30% to ~80%. It seems that further works in this field should be focused on the improvement of tensile properties of studied materials because styrene-butadiene rubber/GTR blends showed tensile strength in the range of 0.5–3.3 MPa.

Toczek, Lipińska, and Pietrasik [284] indicated that selectively chosen thermoplastic elastomers could obtain intelligent materials with shape memory effects. The authors studied the possibility of applying ethylene-1-octene as a binder for recycled EPDM and showed that shape recovery for such systems could reach 87%.

In work [285], studies about GTR modified by ethylene-vinyl acetate copolymer were presented. The main findings showed that the use of a relatively low amount of ethylene-vinyl acetate copolymer improves the mechanical properties of modified reclaimed GTR and also allows further recycling by multiple processing without the deterioration of performance after three cycles, as presented in Table 9.

#### 4.2.2. 3D Printable Materials

3D printing of polymer composites or blends modified by GTR is a relatively new field of research. The first attempts on the application of recycled plastics and waste tire rubber in additive manufacturing technology were described by Domingues et al. [286] in 2017. The authors prepared the recycled polypropylene/ground tire rubber blends 40/60 wt.% using a co-rotating twin-screw extruder, which was characterized by tensile strength ~6 MPa. The prepared blend was used for 3D printing by fused deposition modeling (FDM), replacing the conventional axes system with six degrees of freedom robot. FDM printing parameters were: a layer thickness—10 mm, a deposition velocity—10 mm/s, and extrusion flow rate—3 kg/h. The temperature of the extruder nozzle was around 200 °C, while the temperature of the printing base was 120 °C. This research work confirmed that it is possible to do 3D printing of large parts made by thermoplastics/ground tire rubber blends (with a relatively high content of GTR—60 wt.%).

Alkadi et al. [287] studied the possibility of using GTR as a modifier (used in the range of 50–70 phr) of rubber-like translucent photopolymer (Tangoplus FLX 930 from Stratasys Ltd. Rehovot, Israel) dedicated to Polyjet—multi-material 3D printing. This study showed that the application of GTR modified by 3-(trimethoxysily)propyl methacrylate resulted in better performance properties of 3D printed composites compared to untreated GTR. In conclusion, the authors highlighted directions for further development of this technique, which should focus on improving mechanical properties using different coupling agents and increasing the amount of GTR.

**Figure 6:**
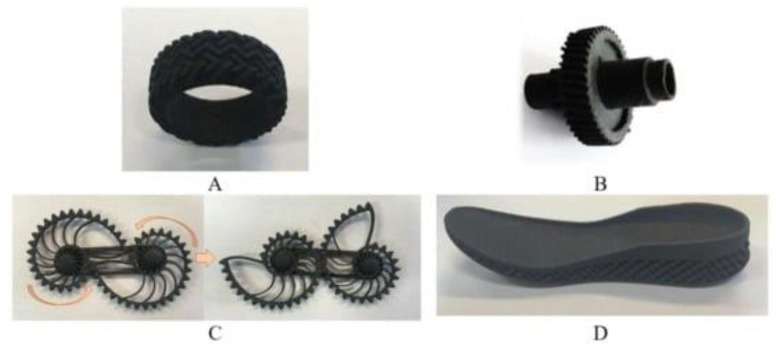
Prototypes 3D printed by selective laser sintering technique: (**A**–**C**) tire and complex shape parts based on PA12 with 20 wt.% of ground tire rubber (particle size below 150 μm) and (**D**) porous flexible insole based on thermoplastic polyurethane (TPU) with 20 wt.% of ground tire rubber (particle size below 150 μm) [288].

Recently, published works [288,289] summarized the results of project VALPREMA-3D, which aimed at utilizing ground tire rubber as a starting component for the production of multifunctional materials dedicated for 3D printing. The examples of prototypes 3D printed by selective laser sintering technique of polyamide 12 modified with 20 wt.% of GTR are presented in Figure 6.

## 5. Conclusions, Limitations, and Future Perspectives

The idea of sustainable development plays a significant role in shaping the way of thinking about mutual relations between the economy, society, and natural resources.

This review presents the recent scientific achievements in the sustainable development of polymeric blends and composites. It can be concluded that the trend of the conscious use of the stream of various post-production and post-consumer wastes is a possibility to reduce the cost of the polymeric materials and as a waste-based additive that can effectively create the desired performance properties of composites.

Grinding processes of the polymeric materials (or other wastes) make it possible to obtain fine particles fractions with relatively low-energy consumption, thanks to the development of novel technologies. The main issue is their separation, which can be done by differences in density in the first step. The still developed methods of electrostatic separation allow for increasing the effectiveness of this method. Nevertheless, it should be emphasized that the growing share of polymer composites and mixtures significantly hinders their identification and separation in the waste stream or after initial grinding and fractionation.

Many literature data have shown that the addition of waste-based additives into polymer matrix usually results in the deterioration of mechanical properties. However, it seems that suitable treatment, modification, or functionalization of used wastes, allows maintaining performance properties similar to the reference material without waste-based additives or in an amount that would exclude the final product in commercial applications.

Moreover, modification of polymeric materials by various waste materials is in line with the climate-energy policy, the aim of which is a reduction of greenhouse gases emissions by limitation of raw materials use or by special features of prepared polymer blends or composites, which reduces their impact on the environment (e.g., biodegradable materials, thermal isolation materials, and waste-based materials).

Future perspectives for development in this field of research should be focused on suitable legislative protocols and promotion activities, resulting in higher awareness within society and finding ways to enforce this on the industry, which represents responsibility for utilization of products after life cycle. New strategies in sustainable development should also consider reducing the number of polymers used in selected industries, such as minimizing the share of multi-material disposable food packaging. However, it should be pointed out that a large part of the products in actual use, for example, in the multi-material injection technology used in automotive or wind turbine blades, pose significant problems in management, separation, and further recycling. Analysis of their further separation may show that it will not be reasonable from an economic and environmental point of view, taking into account the increased complexity of the processing procedures. Therefore, one of the perspectives of processing difficult-to-separate waste is the search for new compatibilization strategies combined with highly efficient processes, especially those based on continuous processes (e.g., reactive extrusion), which allow for more straightforward implementation of results from the laboratory to an industrial scale.

On the other hand, the biggest objection to all polymeric materials made with a share of various wastes is the lack of constancy of the chemical composition and usually very limited characterization (or even incorrect) of waste material before its application. Many works, showing satisfactory results in the laboratory scale, often do not mention process repeatability or the main drawbacks of the proposed solutions (e.g., application of time-consuming or multi-step protocols for material preparation, exceeded use of solvents, lack of information about energy consumption), which seems to be the main limitation for further development and upscaling of waste-based polymer materials:

## Figures and Tables

**Table 1:** Comparison of parameters of cryogenic and various ambient grinding technology of waste truck tires (data adopted from [24]).

Parameter	Grinding Technology			
	Cryogenic	Solid-State Shear Extrusion	Wet Grinding	Hyperboloidal Cutting Mill
Average energy demand (kW)	138	165	32	1.2
Throughput (kg/h)	588	53	61	1.2
Energy consumption (Wh/kg)	1207	3132	525	967

**Table 2:** The main approaches in “Sustainable Plastics Strategy” proposed by SUSCHEM (based on information from [28]).

Sustainable Plastics Strategy		
Sustainable-By-Design	Sustainable Recycling	Alternative Feedstock
Material design: - extend lifetime - higher performance - biodegradability - higher recyclability - limitation of micro- and nano-plastics generated to the environment	Plastic waste pre-treatment: - washing - odor and inks removal	Agricultural and forest biomass waste-based raw materials
	Plastic waste preparation: - grinding technologies - detection systems for ground particles size control	
Article design: - decrease of material usage - decrease of product weight - easier the dismantling of products - PET refillable products (resistant for high temperature washing)	Sorting and separation: - reduction of costs - higher selectivity - higher efficiency	Technologies able to convert CO_2_ and/or CO into polymers or building blocks which can in turn be converted into polymers
	Recycling technologies: - pyrolysis - gasification - depolymerization/solvolysis - dissolution of multi-polymer systems - mechanical recycling	
	Post-processing (actions focused on decontamination of the recycled polymers): - reduction of odor (e.g., barriers/encapsulation) - enhancement of performance (e.g., using of modifiers or compatibilizers)	

**Table 3:** Popularity of polymer matrices used in research about wood polymer composites based on data collected from the Scopus^®^ database (data available on 30 January 2022).

Polymer Matrix	Percent of Works Published in Scopus^®^ in 2016–2022 (%)
PE (bio-PE)	30.1
PP	19.8
PLA	7.8
PVC	5.2
PS and ABS	4.0
PHA and PHB	2.8
PA	1.4
PET	1.3
PCL	1.0
PBAT	0.4
PPC	0.1
Others (mainly thermosets)	26.1

**Table 4:** Humidity and thermal stability of selected natural fibers after various treatment methods (data adopted from [57]).

Fiber Kind	Humidity and TGA * Data	Surface Treatment Method				
		Untreated	Silanization	Acetylation	Benzoylation	Mercerization
Hemp	Humidity (%)	6.5	4.0	6.0	3.0	3.9
	T_-10%_ (°C)	318.9	320.0	289.6	320.4	332.6
	T_-50%_ (°C)	366.1	360.1	342.7	357.4	360.3
	Char residue (%)	11.3	12.5	7.0	10.1	11.7
Flax	Humidity (%)	7.0	5.4	6.0	4.4	5.4
	T_-10%_ (°C)	251.4	318.1	301.8	312.5	264.8
	T_-50%_ (°C)	356.2	363.3	345.1	346.6	358.1
	Char residue (%)	15.4	18.2	5.7	7.1	7.1
Cotton	Humidity (%)	7.5	3.0	4.0	4.0	6.0
	T_-10%_ (°C)	284.7	322.4	316.3	312.7	287.2
	T_-50%_ (°C)	344.4	361.9	350.0	349.9	349.1
	Char residue (%)	13.5	3.8	0.7	0.1	6.7

* TGA: thermogravimetric analysis.

**Table 5:** Processing parameters of poly(lactic acid) green composites filled with linseed cake (data adopted from [89]).

Material	Maximum Torque (Nm)	Torque at 300 s (Nm)	Plasticization Energy (kJ)	MFI_200__°__C/2.16 kg_ (g/10 min)
PLA	57.8	9.9	15.5	10.2
PLA + 5 wt.% of linseed cake	46.2	9.5	15.0	27.0
PLA + 10 wt.% of linseed cake	49.8	6.3	10.2	31.6
PLA + 20 wt.% of linseed cake	28.0	2.3	4.1	127.0
PLA + 30 wt.% of linseed cake	23.4	0.8	2.1	141.0
PLA + 40 wt.% of linseed cake	18.4	0.1	1.2	278.5
PLA + 5 wt.% of linseed cake defatted	43.7	9.6	15.3	23.0
PLA + 10 wt.% of linseed cake defatted	45.4	5.3	9.9	33.0
PLA + 20 wt.% of linseed cake defatted	23.5	4.3	7.3	88.0
PLA + 30 wt.% of linseed cake defatted	21.1	1.6	2.7	195.1
PLA + 40 wt.% of linseed cake defatted	21.8	1.1	3.3	218.7

**Table 6:** Comparison of bio-polyols from plants or waste used in PUR foams dedicated for thermal insulation applications.

Raw Materials	Synthesis Method	LOH, mg KOH/g	M_n_, g/mol	η, mPa·s	%BioP, php	References
Extracts from seeds of *Colliguaja integerrima* and *Colliguaja salicifolia*	Single-step reaction using a mixture of hydrogen peroxide and acetic acid	225; 241	1122; 1166	3637; 5746 (mm^2^/s)	na	[121]
Used cooking oil	Transesterification with diethylene glycol or triethanolamine	277; 348	492; 522	56; 182	20–100	[123]
	Epoxidation and opening oxirane rings with diethylene glycol	150	2557	na	20	[127]
	Epoxidation and opening oxirane rings with diethylene glycol	140; 159	250	961; 3275	20–100	[143]
Biomass from forest waste	Liquefaction with PEG400 and/or glycerol	238–815	na	na	90	[131,132]
Walnut shells	Liquefaction with PEG400 and glycerol	340	420	2550	10–30	[133]
Starch	Reaction of starch with propylene carbonate or ethylene carbonate in aqueous solution	275; 323	-	17,956; 19,058	100	[134]
Cellulose	Hydroxyalkylation with glycidol and ethylene carbonate	688	1650	5538	na	[135]
Tall oil	Epoxidation and opening oxirane rings followed by esterification reactions with different polyfunctional alcohols: trimethylolpropane and triethanolamine	335–519	893–2112	7400–278,300	85	[138]
Waste PLA	Transesterification with diethylene glycol	210–262	341–414	2459–8681	15–62	[142]

LOH—hydroxyl value; M_n_—number molecular weight; η**—**viscosity; %BioP—content of bio-polyol in polyol premix.

**Table 7:** Selected examples of main findings and observation in recently developed green PUR foams.

Filler	Filler Content	Biopolyol	Main Findings and Observations	References
Cellulose	1–3 php	Rapeseed oil-based polyol	- increase in the cell density and reduction of cell sizes	[161]
Solid waste generated in leather industry	0.1–5 php	no	- higher apparent density (0.1 php) - improved compression strength (0.1 php) - less water uptake - addition of filler over a certain optimal level has a negative effect on the cell morphology and physico-mechanical properties	[162]
Potato protein	0.1–5 php	no	- improved compressive strength (0.1 and 1 php)	[163]
Walnut shells silanized	1–5 php	no	- improved physical-mechanical properties, - improved thermal insulating properties - (silanized walnut shells 1 php)	[164]
Cloves	1, 2, and 5 wt.%	Soybean oil-based polyol	- improved compression strength - improved flexural strength - improved impact strength - improved antibacterial properties	[165]
Walnut shells (unmodified and treated)	2 php	Walnut shells-based polyol	- reduction of cell size - improved compressive strength - slight deterioration of the thermal conductivity coefficient	[166]
Hemp shives and impregnated hemp shives	2 php	no	- improved compressive strength for materials modified with non-treated hemp shives - improved thermal stability and flame retardancy of materials modified with impregnated hemp shives	[167]
Nutmeg	1–5 wt.%	no	- improved compression strength (1 wt.%) - higher flexural strength (1 wt.%) - improved impact strength (1 wt.%) - positive effect on the fire resistance (5 wt.%)	[170]
By-product from vegetable oil industry–rapeseed cake	30–60 wt.%	no	- lower reactivity - increased apparent density - no significant effect on thermal conductivity - tendency for opening the cells - smaller cross-sectional area of cells - higher compressive strength - lower brittleness - lower flammability	[171]
Egg shells	20 php	Rapeseed oil-based polyol 10–50 php	- the compressive strength was unaffected by the introduction of egg shells for materials unmodified with bio-polyol and modified in an amount of 10 php	[172]
Biomass incineration waste ash	10–50 php	Rapeseed oil-based polyol 60 php	- higher apparent density - improved compressive strength (10 and 30 php) - improved thermal stability - reduced average heat release during cone calorimeter test	[173]
Eucalyptus fibers (unmodified and treated)	2 php	no	- improved mechanical and thermal properties	[174]

**Table 8:** Ground tire rubber fractions offered by Grupa Recykl S.A. (data adopted from [226]).

Particle Size (mm)	Percentage Content (%) *							
	GTR 4–7	GTR 2–6	GTR 1–4	GTR 1–3	GTR 0.5–2.5	GTR 0.0–2.5	GTR 0.5–2.0	GTR 0.3–1.5
8.0	0.0	0.0	0.0	0.0	0.0	0.0	0.0	0.0
7.0	0.0	0.0	0.0	0.0	0.0	0.0	0.0	0.0
6.0	8.1	5.2	0.0	0.0	0.0	0.0	0.0	0.0
5.0	24.0	18.5	0.0	0.0	0.0	0.0	0.0	0.0
4.0	41.7	33.8	0.6	0.0	0.0	0.0	0.0	0.0
3.0	22.8	28.1	23.1	0.6	0.7	0.0	0.0	0.0
2.0	3.4	14.3	59.2	59.1	54.6	8.5	6.5	17.8
1.0	0.0	0.1	17.1	40.3	43.7	80.1	83.1	39.9
<1.0	0.0	0.0	0.0	0.0	1.0	11.4	10.4	42.3

* residue after sieving.

**Table 9:** Mechanical properties and appearance of modified GTR as function of recycling cycles (adopted from [285]).

Property	Standard	Reference	Recycling Step		
			1st	2nd	3rd
Tensile strength (MPa)	ISO 37	3.2 ± 0.5	2.9 ± 0.4	3.1 ± 0.4	2.7 ± 0.5
Elongation at break (%)	ISO 37	146 ± 11	136 ± 17	143 ± 17	123 ± 25
Hardness (Shore A)	ISO 7619-1	63 ± 1	63 ± 1	64 ± 1	63 ± 1
Appearance of sample	Digital camera	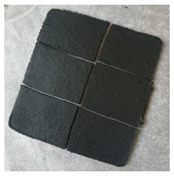	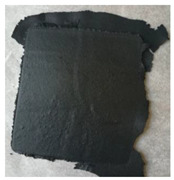		

## Data Availability

Not applicable.

## References

[1] Hsissou R., Seghiri R., Benzekri Z., Hilali M., Rafik M., Elharfi A. (2021). Polymer composite materials: A comprehensive review. Compos. Struct..

[2] Arefin A.M.E., Khatri N.R., Kulkarni N., Egan P.F. (2021). Polymer 3D printing review: Materials, process, and design strategies for medical applications. Polymers.

[3] Formela K., Zedler Ł., Hejna A., Tercjak A. (2018). Reactive extrusion of bio-based polymer blends and composites—Current trends and future developments. Express Polym. Lett..

[4] Wong K.K., Jawad Z.A. (2019). A review and future prospect of polymer blend mixed matrix membrane for CO_2_ separation. J. Polym. Res..

[5] Roy K., Debnath S.C., Potiyaraj P. (2020). A review on recent trends and future prospects of lignin based green rubber composites. J. Polym. Environ..

[6] Tiseo I. Global Plastic Market Size 2016–2028. https://www.statista.com/statistics/1060583/global-market-value-of-plastic/.

[7] Tiseo I. Annual Production of Plastics Worldwide from 1950 to 2020. https://www.statista.com/statistics/282732/global-production-of-plastics-since-1950/.

[8] PlasticsEurope (the Association of Plastics Manufacturers in Europe) Plastics—The Facts 2020. An analysis of European Plastics Production, Demand and Waste Data. https://www.plasticseurope.org.

[9] Yoshida A. (2022). China’s ban of imported recyclable waste and its impact on the waste plastic recycling industry in China and Taiwan. J. Mater. Cycles Waste Manag..

[10] Wen Z., Xie Y., Chen M., Dinga C.D. (2021). China’s plastic import ban increases prospects of environmental impact mitigation of plastic waste trade flow worldwide. Nat. Commun..

[11] Żenkiewicz M., Żuk T. (2014). Physical basis of tribocharging and electrostatic separation of plastics. Polimery.

[12] Żenkiewicz M., Żuk T. (2014). Characteristics of separators and some limitations for electrostatic separation of polymer blends. Przem. Chem..

[13] Żenkiewicz M., Żuk T., Pietraszek J., Rytlewski P., Moraczewski K., Stepczyńska M. (2016). Electrostatic separation of binary mixtures of some biodegradable polymers and poly(vinyl chloride) or poly(ethylene terephthalate). Polimery.

[14] Żuk T., Pietraszak J., Żenkiewicz M. (2016). Modeling of electrostatic separation process for some polymer mixtures. Polimery.

[15] Czarnecka-Komorowska D., Kanciak W., Barczewski M., Barczewski R., Regulski R., Sędziak D., Jędryczka C. (2021). Recycling of plastics from cable waste from automotive industry in Poland as an approach to the circular economy. Polymers.

[16] Lyskawinski W., Baranski M., Jedryczka C., Mikolajewicz J., Regulski R., Sedziak D., Netter K., Rybarczyk D., Czarnecka-Komorowska D., Barczewski M. (2021). Tribo-electrostatic separation analysis of a beneficial solution in the recycling of mixed poly (ethylene terephthalate) and high-density polyethylene. Energies.

[17] Froelich D., Maris E. (2010). Sorting mixed polyolefins from end-of-life product by a selective grinding process. Waste Biomass Valor..

[18] Al-Zubiedy A.A.A. (2019). Analysis of dimensional stability of recycled plastic material obtained by grinding. Polimery.

[19] Flizikowski J. (1998). Rozdrabnianie Tworzyw Sztucznych.

[20] Flizikowski J., Wełnowski J., Dudziak P. (2017). Analysis and eco-technological evaluation of micro-grain supersonic milling. Part I. Models and indicators. Inż. Ap. Chem..

[21] Flizikowski J., Wełnowski J., Dudziak P. (2017). Analysis and eco-technological evaluation of micro-grain supersonic milling. Part II. Results and discussion. Inż. Ap. Chem..

[22] Hejna A. (2021). Clays as inhibitors of polyurethane foams’ flammability. Materials.

[23] Spina R., Cavalcante B. (2020). Evaluation of grinding of unfilled and glass fiber reinforced polyamide 6,6. Polymers.

[24] Hoyer S., Kroll L., Sykutera D. (2020). Technology comparison for the production of fine rubber powder from end of life tyres. Procedia Manuf..

[25] Guo X., Xiang D., Duan G., Mou P. (2010). A review of mechanochemistry applications in waste management. Waste Manag..

[26] Cichosz S., Masek A. (2020). Superiority of cellulose non-solvent chemical modification over solvent-involving treatment: Solution for green chemistry (Part I). Materials.

[27] Bula K., Klapiszewski Ł., Piasecki A., Jesionowski T. (2021). The role of inorganic-organic bio-fillers containing kraft lignin in improvement in functional properties of polyethylene. Materials.

[28] Sustainable Plastics Strategy. http://www.suschem.org/publicationsbySUSCHEM.

[29] Zaaba N.F., Ismail H. (2019). Thermoplastic/natural filler composites: A short review. J. Phys. Sci..

[30] Thomason J.L., Rudeiros-Fernández J.L. (2018). A review of the impact performance of natural fiber thermoplastic composites. Front. Mater..

[31] Wahit M.U., Akos N.I., Laftah W.A. (2012). Influence of natural fibers on the mechanical properties and biodegradation of poly (lactic acid) and poly (ε-caprolactone) composites: A review. Polym. Compos..

[32] Raport Plastics—The Facts 2021 from PlasticEurope. https://plasticseurope.org/pl/knowledge-hub/plastics-the-facts-2021/.

[33] https://www.european-bioplastics.org/market/.

[34] Aniśko J., Barczewski M. (2021). Polylactide: From synthesis and modification to final properties. Adv. Sci. Technol. Res. J..

[35] Thomas R.J. (1977). Wood: Structure and Chemical Composition in Book: Wood Technology: Chemical Aspects.

[36] Kozlowski R., Wladyka-Przybylak M., Horrocks A.R., Price D. (2001). Natural Polymers, wood and lignocellulosic materials. Fire Retardant Materials.

[37] Reszka P. (2008). In-Depth Temperature Profiles in Pyrolzing Wood.

[38] Olabisi O., Adewale K. (2016). Handbook of Thermoplastics.

[39] Pradhan R., Misra M., Erickson L., Mohanty A. (2010). Compostability and biodegradation study of PLA–wheat straw and PLA–soy straw based green composites in simulated composting bioreactor. Bioresour. Technol..

[40] Dilucia F., Lacivita V., Conte A., del Nobile M.A. (2020). Sustainable use of fruit and vegetable by-products to enhance food packaging performance. Foods.

[41] Efenberger-Szmechtyk M., Nowak A., Czyzowska A. (2021). Plant extracts rich in polyphenols: Antibacterial agents and natural preservatives for meat and meat products. Crit. Rev. Food Sci. Nutr..

[42] Shah A.U.R., Prabhakar M.N., Song J.-I. (2017). Current advances in the fire retardancy of natural fiber and bio-based composites—A review. Int. J. Precis. Eng. Manuf. Technol..

[43] Ashori A., Nourbakhsh A. (2010). Bio-based composites from waste agricultural residues. Waste Manag..

[44] Nagarajan V., Mohanty A.K., Misra M. (2013). Sustainable green composites: Value addition to agricultural residues and perennial grasses. ACS Sustain. Chem. Eng..

[45] Song X., He W., Chen P., Wei Q., Wen J., Xiao G. (2021). Fused deposition modeling of poly (lactic acid)/almond shell composite filaments. Polym. Compos..

[46] Altun M., Celebi M., Ovali S. (2021). Preparation of the pistachio shell reinforced PLA biocomposites: Effect of filler treatment and PLA maleation. J. Thermoplast. Compos. Mater..

[47] Odili C., Gbenebor O., Adesola O.E., Adeosun S. (2021). Characterization of polylactide (PLA) composite reinforced with biowaste. Kufa. J. Eng..

[48] Hejna A., Sulyman M., Przybysz M., Saeb M.R., Klein M., Formela K. (2020). On the correlation of lignocellulosic filler composition with the performance properties of poly(ε-caprolactone) based biocomposites. Waste Biomass Valor..

[49] Mirowski J., Oliwa R., Oleksy M., Tomaszewska J., Ryszkowska J., Budzik G. (2021). Poly (vinyl chloride) composites with raspberry pomace filler. Polymers.

[50] Sałasińska K., Osica A., Ryszkowska J. (2012). The use of tree leaves as reinforcement in composites with recycled PE-HD matrix. Polimery.

[51] Kengkhetkit N., Amornsakchai T. (2014). A new approach to “greening” plastic composites using pineapple leaf waste for performance and cost effectiveness. Mater. Des..

[52] Kufel A., Kuciel S. (2019). Composites based on polypropylene modified with natural fillers to increase stiffness. Czas. Tech..

[53] Stark N.M., Rowlands R.E. (2003). Effects of wood fiber characteristics on mechanical properties of wood/polypropylene composites. Wood Fiber Sci..

[54] Amir N., Abidin K.A.Z., Shiri F.B.M. (2017). Effects of fibre configuration on mechanical properties of banana fibre/PP/MAPP natural fibre reinforced polymer composite. Procedia Eng..

[55] Dun M., Fu H., Hao J., Shan W., Wang W. (2021). Tailoring flexible interphases in bamboo fiber-reinforced linear low-density polyethylene composites. Compos. Part A Appl. Sci. Manuf..

[56] Sherwani S., Sapuan S., Leman Z., Zainudin E., Khalina A. (2022). Effect of alkaline and benzoyl chloride treatments on the mechanical and morphological properties of sugar palm fiber-reinforced poly (lactic acid) composites. Text. Res. J..

[57] Matykiewicz D., Barczewski M., Mysiukiewicz O., Skórczewska K. (2021). Comparison of various chemical treatments efficiency in relation to the properties of flax, hemp fibers and cotton trichomes. J. Nat. Fibers.

[58] Rojas-Lema S., Lascano D., Ivorra-Martinez J., Gomez-Caturla J., Balart R., Garcia-Garcia D. (2021). Manufacturing and Characterization of high-density polyethylene composites with active fillers from persimmon peel flour with improved antioxidant activity and hydrophobicity. Macromol. Mater. Eng..

[59] Barczewski M., Matykiewicz D., Piasecki A., Szostak M. (2018). Polyethylene green composites modified with post agricultural waste filler: Thermo-mechanical and damping properties. Compos. Interfaces.

[60] Paukszta D., Doczekalska B., Ostrowski A., Bartkowiak M. (2015). Modification of rapeseed straw with organic acid anhydrides. J. Compos. Mater..

[61] Hejna O., Marć M., Korol J. (2021). Modification of cellulosic filler with diisocyanates–volatile organic compounds emission assessment and stability of chemical structure over time. Nord. Pulp Pap. Res. J..

[62] Hejna A., Barczewski M., Skórczewska K., Szulc J., Chmielnicki B., Korol J., Formela K. (2021). Sustainable upcycling of brewers’ spent grain by thermo-mechanical treatment in twin-screw extruder. J. Clean. Prod..

[63] Rytlewski P., Moraczewski K., Malinowski R., Żenkiewicz M. (2014). Assessment of dicumyl peroxide ability to improve adhesion between polylactide and flax or hemp fibres. Compos. Interfaces.

[64] Luo S., Cao J., Armando G., McDonald A.G. (2016). Interfacial improvements in a green biopolymer alloy of polyn (3-hydroxybutyrate-co-3-hydroxyvalerate) and lignin via in situ reactive extrusion. ACS Sustain. Chem. Eng..

[65] Dhar P., Gaur S.S., Soundararajan N., Gupta A., Bhasney S.M., Milli M., Kumar A., Katiyar V. (2017). Reactive extrusion of polylactic acid/cellulose nanocrystal films for food packaging applications: Influence of filler type on thermomechanical, rheological, and barrier properties. Ind. Eng. Chem. Res..

[66] Reinaldo J.S., Milfont C.H.R., Gomes F.P.C., Mattos A.L.A., Medeiros F.G.M., Lopes P.F.N., Filho M., de Sá M.S., Matsui K.N., Ito E.N. (2021). Influence of grape and acerola residues on the antioxidant, physicochemical and mechanical properties of cassava starch biocomposites. Polym. Test..

[67] Napolitano A., Fogliano V., Tafuri A., Ritieni A. (2007). Natural occurrence of ochratoxin A and antioxidant activities of green and roasted coffees and corresponding byproducts. J. Agric. Food Chem..

[68] Battegazzore D., Bocchini S., Alongi J., Frache A. (2014). Plasticizers, antioxidants and reinforcement fillers from hazelnut skin and cocoa by-products: Extraction and use in PLA and PP. Polym. Degrad. Stab..

[69] Gaur A., Tiwari S., Kumar C., Maiti P. (2020). Bio-waste orange peel and polymer hybrid for efficient energy harvesting. Energy Rep..

[70] Hejna A. (2021). Potential applications of by-products from the coffee industry in polymer technology—Current state and perspectives. Waste Manag..

[71] Oliveira G., Passos C.P., Ferreira P., Coimbra M.A., Gonçalves I. (2021). Coffee by-products and their suitability for developing active food packaging materials. Foods.

[72] Janissen B., Huynh T. (2018). Chemical composition and value-adding applications of coffee industry by-products: A review. Resour. Conserv. Recycl..

[73] Totaro G., Sisti L., Fiorini M., Lancellotti I., Andreola F.N., Saccani A. (2019). Formulation of green particulate composites from PLA and PBS matrix and wastes deriving from the coffee production. J. Polym. Environ..

[74] Sarasini F., Luzi F., Dominici F., Maffei G., Iannone A., Zuorro A., Lavecchia R., Torre L., Carbonell-Verdu A., Balart R. (2018). Effect of different compatibilizers on sustainable composites based on a PHBV/PBAT matrix filled with coffee silverskin. Polymers.

[75] Kumar T.S.M., Rajini N., Huafeng T., Rajulu A.V., Ayrilmis N., Siengchin S. (2019). Improved mechanical and thermal properties of spent coffee bean particulate reinforced poly (propylene carbonate) composites. Part. Sci. Technol..

[76] Iyer K.A., Zhang L., Torkelson J.M. (2016). Direct use of natural antioxidant-rich agro-wastes as thermal stabilizer for polymer: Processing and recycling. ACS Sustain. Chem. Eng..

[77] Nanni A., Battegazzore D., Frache A., Messori M. (2019). Thermal and UV aging of polypropylene stabilized by wine seeds wastes and their extracts. Polym. Degrad. Stab..

[78] Moraczewski K., Karasiewicz T., Jagodziński B., Trafarski A., Pawłowska A., Stepczyńska M., Rytlewski P. (2021). Recyclability of new polylactide based biodegradable materials with plant extracts containing natural polyphenols. Sustain. Mater. Technol..

[79] Jóźwik A., Strzałkowska N., Markiewicz-Keszycka M., Krzyżewski J., Lipińska P., Rutkowska J., Wróblewska B., Klusek J., Cooper R. (2016). Effects of replacing rapeseed cake with linseed cake in a corn-grass silage-based diet for milking cows. Anim. Sci. Pap. Rep..

[80] Łopusiewicz Ł., Drozłowska E., Tarnowiecka-Kuca A., Bartkowiak A., Mazurkiewicz-Zapałowicz K., Salachna P. (2020). Biotransformation of flaxseed oil cake into bioactive camembert-analogue using lactic acid bacteria, Penicillium camemberti and Geotrichum candidum. Microorganisms.

[81] Łopusiewicz Ł., Drozłowska E., Siedlecka P., Mężyńska M., Bartkowiak A., Sienkiewicz M., Zielińska-Bliźniewska H., Kwiatkowski P. (2019). Development, characterization, and bioactivity of non-dairy kefir-like fermented beverage based on flaxseed oil cake. Foods.

[82] Arrieta M.P., Samper M.D., Jiménez-López M., Aldas M., López J. (2017). Combined effect of linseed oil and gum rosin as natural additives for PVC. Ind. Crops Prod..

[83] Fenollar O., Garcia-Sanoguera D., Sanchez-Nacher L., Lopez J., Balart R. (2010). Effect of the epoxidized linseed oil concentration as natural plasticizer in vinyl plastisols. J. Mater. Sci..

[84] Garcia-Campo M., Quiles-Carrillo L., Masia J., Reig-Pérez M., Montanes N., Balart R. (2017). Environmentally friendly compatibilizers from soybean oil for ternary blends of poly (lactic acid)-PLA, poly (ε-caprolactone)-PCL and poly (3-hydroxybutyrate)-PHB. Materials.

[85] Quiles-Carrillo L., Duart S., Montanes N., Torres-Giner S., Balart R. (2018). Enhancement of the mechanical and thermal properties of injection-molded polylactide parts by the addition of acrylated epoxidized soybean oil. Mater. Des..

[86] Mittal V., Luckachan G.E., Chernev B., Matsko N.B. (2015). Bio-polyester-date seed powder composites: Morphology and component migration. Polym. Eng. Sci..

[87] Mysiukiewicz O., Barczewski M. (2019). Utilization of linseed cake as a postagricultural functional filler for poly (lactic acid) green composites. J. Appl. Polym. Sci..

[88] Mysiukiewicz O., Barczewski M. (2020). Crystallization of polylactide-based green composites filled with oil-rich waste fillers. J. Polym. Res..

[89] Barczewski M., Mysiukiewicz O., Szulc J., Kloziński A. (2019). Poly (lactic acid) green composites filled with linseed cake as an agricultural waste filler. Influence of oil content within the filler on the rheological behavior. J. Appl. Polym. Sci..

[90] Kozłowski R., Władyka-Przybylak M. (2008). Flammability and fire resistance of composites reinforced by natural fibers. Polym. Adv. Technol..

[91] John M.J., Koronis G., Silva A. (2018). Chapter 2: Flammability performance of biocomposites. Green Composites for Automotive Applications.

[92] Duarah P., Haldar D., Purkait M.K. (2020). Technological advancement in the synthesis and applications of lignin-based nanoparticles derived from agro-industrial waste residues: A review. Int. J. Biol. Macromol..

[93] Yu Y., Fu S., Song P., Luo X., Jin Y., Lu F., Wu Q., Ye J. (2012). Functionalized lignin by grafting phosphorus-nitrogen improves the thermal stability and flame retardancy of polypropylene. Polym. Degrad. Stab..

[94] Wang Y., Yue J., Xie R., Liu C., Gan L., Huang J. (2020). High-value use of lignocellulosic-rich eucommia residue for promoting mechanical properties and flame retardancy of poly (butylene succinate). J. Appl. Polym. Sci..

[95] Sałasińska K., Barczewski M., Borucka M., Górny R.L., Kozikowski P., Celiński M., Gajek A. (2019). Thermal stability, fire and smoke behaviour of epoxy composites modified with plant waste fillers. Polymers.

[96] Sałasińska K., Celiński M., Mizera K., Kozikowski P., Leszczyński M.K., Gajek A. (2020). Synergistic effect between histidine phosphate complex and hazelnut shell for flammability reduction of low-smoke emission epoxy resin. Polym. Degrad. Stab..

[97] Sałasińska K., Mizera K., Barczewski M., Borucka M., Gloc M., Celiński M., Gajek A. (2019). The influence of degree of fragmentation of Pinus sibirica on flammability, thermal and thermomechanical behavior of the epoxy-composites. Polym. Test..

[98] Vahabi H., Jouyandeh M., Parpaite T., Saeb M.R., Ramakrishna S. (2021). Coffee wastes as sustainable flame retardants for polymer materials. Coatings.

[99] Hajj R., el Hage R., Sonnier R., Otazaghine B., Gallard B., Rouif S., Nakhl M., Lopez-Cuesta J.M. (2018). Grafting of phosphorus flame retardants on flax fabrics: Comparison between two routes. Polym. Degrad. Stab..

[100] le Moigne N., Sonnier R., El Hage R., Rouif S. (2017). Radiation-induced modifications in natural fibres and their biocomposites: Opportunities for controlled physico-chemical modification pathways?. Ind. Crops Prod..

[101] Al Hokayem K., El Hage R., Svecova L., Otazaghine B., le Moigne N., Sonnier R. (2020). Flame retardant-functionalized cotton cellulose using phosphonate-based ionic liquids. Molecules.

[102] Oatway L., Vasanthan T., Helm J.H. (2001). Phytic acid. Food Rev. Int..

[103] Yang W., Tawiah B., Yu C., Qian Y.-F., Wang L.-L., Yuen C.-Y.A., Zhu S.-E., Hu E.-Z., Chen B.-Y.T., Yu B. (2018). Manufacturing, mechanical and flame retardant properties of poly (lactic acid) biocomposites based on calcium magnesium phytate and carbon nanotubes. Compos. Part A Appl. Sci..

[104] Costes L., Laoutid F., Dumazert L., Lopez-Cuesta J.M., Brohez S., Delvosalle C., Dubois P. (2015). Metallic phytates as efficient bio-based phosphorous flame retardant additives for poly (lactic acid). Polym. Degrad. Stab..

[105] Yang Y.X., Haurie L., Zhang J., Zhang X.-Q., Wang R., Wang D.-Y. (2020). Effect of bio-based phytate (PA-THAM) on the flame retardant and mechanical properties of polylactide (PLA). Express Polym. Lett..

[106] El-Nemr K.F., Mohamed H.R., Ali M.A., Fathy R.M., Dhmees A.S. (2020). Polyvinyl alcohol/gelatin irradiated blends filled by lignin as green filler for antimicrobial packaging materials. Int. J. Environ. Anal. Chem..

[107] Spiridon I., Anghel N.C., Darie-Nita R.N., Iwańczuk A., Ursu R.G., Spiridon I.A. (2020). New composites based on starch/Ecoflex®/biomass wastes: Mechanical, thermal, morphological and antimicrobial properties. Int. J. Biol. Macromol..

[108] Eswaranandam S., Hettiarachchy N.S., Johnson M.G. (2006). Antimicrobial activity of citric, lactic, malic, or tartaric acids and nisin-incorporated soy protein film against Listeria monocytogenes, Escherichia coli O157:H7, and *Salmonella gaminara*. J. Food Sci..

[109] Spiridon I., Ursu R.G., Spiridon I.A.C. (2015). New polylactic acid composites for packaging applications: Mechanical properties, thermal behavior, and antimicrobial activity. Int. J. Polym. Anal. Charact..

[110] Klapiszewski Ł., Oliwa R., Oleksy M., Jesionowski T. (2018). Calcium lignosulfonate as eco-friendly additive for crosslinking fibrous composites with phenol-formaldehyde resin matrix. Polimery.

[111] Kawalerczyk J., Dziurka D., Mirski R., Trociński A. (2019). Flour fillers with urea-formaldehyde resin in plywood. BioResources.

[112] Pączkowski P., Puszka A., Gawdzik B. (2020). Green composites based on unsaturated polyester resin from recycled poly (ethylene terephthalate) with wood flour as filler—Synthesis, characterization and aging effect. Polymers.

[113] Sienkiewicz A., Czub P. (2019). Blocked isocyanates as alternative curing agents for epoxy-polyurethane resins based on modified vegetable oil. Express Polym. Lett..

[114] Jankowski A., Grabiec E., Nocoń-Szmajda K., Marcinkowski A., Janeczek H., Wolińska-Grabczyk A. (2021). Polyimide-based membrane materials for CO_2_ separation: A comparison of segmented and aromatic (co)polyimides. Membranes.

[115] Głowińska E., Wolak W., Datta J. (2021). Eco-friendly route for thermoplastic polyurethane elastomers with bio-based hard segments composed of bio-glycol and mixtures of aromatic–aliphatic and aliphatic–aliphatic diisocyanate. J. Polym. Environ..

[116] Sardon H., Mecerreyes D., Basterretxea A., Avérous L., Jehanno C. (2021). From lab to market: Current strategies for the production of biobased polyols. ACS Sustain. Chem. Eng..

[117] Borowicz M., Paciorek-Sadowska J., Lubczak J., Czupryński B. (2019). Biodegradable, flame-retardant, and bio-based rigid polyurethane/polyisocyanurate foams for thermal insulation application. Polymers.

[118] Fridrihsone A., Abolins A., Kirpluks M. (2020). Screening life cycle assessment of tall oil-based polyols suitable for rigid polyurethane foams. Energies.

[119] Pietrzak K., Kirpluks M., Cabulis U., Ryszkowska J. (2014). Effect of the addition of tall oil-based polyols on the thermal and mechanical properties of ureaurethane elastomers. Polym. Degrad. Stab..

[120] de Luca Bossa F., Verdolotti L., Russo V., Campaner P., Minigher A., Lama G.C., Boggioni L., Tesser R., Lavorgna M. (2020). Upgrading sustainable polyurethane foam based on greener polyols: Succinic-based polyol and Mannich-based polyol. Materials.

[121] Abril-Milán D., Valdés O., Mirabal-Gallardo Y.F., de la Torre A., Bustamante C., Contreras J. (2018). Preparation of renewable bio-polyols from two species of Colliguaja for rigid polyurethane foams. Materials.

[122] Kurańska M., Beneš H., Prociak A., Trhlíková O., Walterová Z., Stochlińska W. (2019). Investigation of epoxidation of used cooking oils with homogeneous and heterogeneous catalysts. J. Clean. Prod..

[123] Kurańska M., Malewska E., Polaczek K., Prociak A., Kubacka J. (2020). A pathway toward a new era of open-cell polyurethane foams—Influence of bio-polyols derived from used cooking oil on foams properties. Materials.

[124] Kurańska M., Niemiec M. (2020). Cleaner production of epoxidized cooking oil using a heterogeneous catalyst. Catalysts.

[125] Kurańska M., Beneš H., Polaczek K., Trhlikova O., Walterova Z., Prociak A. (2019). Effect of homogeneous catalysts on ring opening reactions of epoxidized cooking oils. J. Clean. Prod..

[126] Kurańska M., Banaś J., Polaczek K., Banaś M., Prociak A., Kuc J., Uram K., Lubera T. (2019). Evaluation of application potential of used cooking oils in the synthesis of polyol compounds. J. Environ. Chem. Eng..

[127] Polaczek K., Kurańska M., Auguścik-Królikowska M., Prociak A., Ryszkowska J. (2021). Open-cell polyurethane foams of very low density modified with various palm oil-based bio-polyols in accordance with cleaner production. J. Clean. Prod..

[128] Serrano L., Rincón E., García A., Rodríguez J., Briones R. (2020). Bio-degradable polyurethane foams produced by liquefied polyol from wheat straw biomass. Polymers.

[129] Wang Q., Tuohedi N. (2020). Polyurethane foams and bio-polyols from liquefied cotton stalk agricultural waste. Sustainability.

[130] Jiang W., Hosseinpourpia R., Biziks V., Ahmed S.A., Militz H., Adamopoulos S. (2021). Preparation of polyurethane adhesives from crude and purified liquefied wood sawdust. Polymers.

[131] Gosz K., Tercjak A., Olszewski A., Haponiuk J., Piszczyk Ł. (2021). Bio-based polyurethane networks derived from liquefied sawdust. Materials.

[132] Olszewski A., Nowak P., Kosmela P., Piszczyk Ł. (2021). Characterization of highly filled glass fiber/carbon fiber polyurethane composites with the addition of bio-polyol obtained through biomass liquefaction. Materials.

[133] Członka S., Strąkowska A., Kairytė A. (2020). Application of walnut shells-derived biopolyol in the synthesis of rigid polyurethane foams. Materials.

[134] Lubczak R., Szczęch D., Broda D., Wojnarowska-Nowak R., Kus-Liśkiewicz M., Dębska B., Lubczak J. (2021). Polyetherols and polyurethane foams from starch. Polym. Test..

[135] Szpiłyk M., Lubczak R., Lubczak J. (2021). The biodegradable cellulose-derived polyol and polyurethane foam. Polym. Test..

[136] Ionescu M., Wan X., Bilić N., Petrović Z.S. (2012). Polyols and rigid polyurethane foams from cashew nut shell liquid. J. Polym. Environ..

[137] Gandhi T.S., Patel M.R., Dholakiya B.Z. (2015). Mechanical, thermal and fire properties of sustainable rigid polyurethane foam derived from cashew nut shell liquid. Int. J. Plast. Technol..

[138] Abolins A., Pomilovskis R., Vanags E., Mierina I., Michalowski S., Fridrihsone A., Kirpluks M. (2021). Impact of different epoxidation approaches of tall oil fatty acids on rigid polyurethane foam thermal insulation. Materials.

[139] Borowicz M., Paciorek-Sadowska J., Isbrandt M., Grzybowski Ł., Czupryński B. (2019). Glycerolysis of poly (lactic acid) as a way to extend the “life cycle” of this material. Polymers.

[140] Datta J., Kopczyńska P. (2016). From polymer waste to potential main industrial products: Actual state of recycling and recovering. Crit. Rev. Environ. Sci. Technol..

[141] Paciorek-Sadowska J., Borowicz M., Isbrandt M. (2019). New poly(lactide-urethane-isocyanurate) foams based on bio-polylactide waste. Polymers.

[142] Borowicz M., Isbrandt M., Paciorek-Sadowska J. (2021). Effect of new eco-polyols based on PLA waste on the basic properties of rigid polyurethane and polyurethane/polyisocyanurate foams. Int. J. Mol. Sci..

[143] Kurańska M., Polaczek K., Auguścik-Królikowska M., Prociak A., Ryszkowska J. (2020). Open-cell rigid polyurethane bio-foams based on modified used cooking oil. Polymer.

[144] Gao T.-Y., Wang F.-D., Xu Y., Wei C.-X., Zhu S.-E., Yang W., Lu H.-D. (2022). Luteolin-based epoxy resin with exceptional heat resistance, mechanical and flame retardant properties. Chem. Eng. J..

[145] Tian Y., Wang Q., Cheng J., Zhang J. (2020). A fully biomass based monomer from itaconic acid and eugenol to build degradable thermosets via thiol–ene click chemistry. Green Chem..

[146] Taung Mai L.L., Aung M.M., Muhamad Saidi S.A., H’ng P.S., Rayung M., Jaafar A.M. (2021). Non edible oil-based epoxy resins from Jatropha oil and their shape memory behaviors. Polymers.

[147] Sienkiewicz A., Czub P., Milo A. (2021). Palm oil as a renewable raw material in the synthesis of new polymeric materials through the epoxy fusion process. Express Polym. Lett..

[148] Sienkiewicz A., Czub P. (2021). Rheological analysis of the synthesis of high-molecular-weight epoxy resins from modified soybean oil and bisphenol A or BPA-based epoxy resins. Materials.

[149] Lascano D., Lerma-Canto A., Fombuena V., Balart R., Montanes N., Quiles-Carrillo L. (2021). Kinetic analysis of the curing process of biobased epoxy resin from epoxidized linseed oil by dynamic differential scanning calorimetry. Polymers.

[150] Sienkiewicz N., Dominic M., Parameswaranpillai J. (2022). Natural fillers as potential modifying agents for epoxy composition: A review. Polymers.

[151] Gioia C., Colonna M., Tagami A., Medina L., Sevastyanova O., Berglund L.A., Lawoko M. (2020). Lignin-based epoxy resins: Unravelling the relationship between structure and material properties. Biomacromolecules.

[152] Roszowska-Jarosz M., Masiewicz J., Kostrzewa M., Kucharczyk W., Żurowski W., Kucińska-Lipka J., Przybyłek P. (2021). Mechanical properties of bio-composites based on epoxy resin and nanocellulose fibres. Materials.

[153] Gargol M., Klepka T., Klapiszewski Ł., Podkościelna B. (2021). Synthesis and thermo-mechanical study of epoxy resin-based composites with waste fibers of hemp as an eco-friendly filler. Polymers.

[154] Hodul J., Mészárosová L., Drochytka R. (2021). Recovery of industrial wastes as fillers in the epoxy thermosets for building application. Materials.

[155] Kurańska M., Polaczek K., Auguścik-Królikowska M., Prociak A., Ryszkowska J. (2020). Open-cell polyurethane foams based on modified used cooking oil. Polimery.

[156] Kurańska M., Barczewski R., Barczewski M., Prociak A., Polaczek K. (2020). Thermal insulation and sound absorption properties of open-cell polyurethane foams modified with bio-polyol based on used cooking oil. Materials.

[157] Kurańska M., Beneš H., Sałasińska K., Prociak A., Malewska E., Polaczek K. (2020). Development and characterization of “green open-cell polyurethane foams” with reduced flammability. Materials.

[158] Kurańska M., Leszczyńska M., Kubacka J., Prociak A., Ryszkowska J. (2020). Effects of modified used cooking oil on structure and properties of closed-cell polyurethane foams. J. Polym. Environ..

[159] Kurańska M., Leszczyńska M., Malewska E., Prociak A., Ryszkowska J. (2020). Implementation of circular economy principles in the synthesis of polyurethane foams. Polymers.

[160] Członka S., Strąkowska A., Strzelec K., Adamus-Włodarczyk A., Kairytė A., Vaitkus S. (2019). Composites of rigid polyurethane foams reinforced with POSS. Polymers.

[161] Uram K., Leszczyńska M., Prociak A., Czajka A., Gloc M., Leszczyński M.K., Michałowski S., Ryszkowska J. (2021). Polyurethane composite foams synthesized using bio-polyols and cellulose filler. Materials.

[162] Członka S., Bertino M.F., Strzelec K., Strąkowska A., Masłowski M. (2018). Rigid polyurethane foams reinforced with solid waste generated in leather industry. Polym. Test..

[163] Członka S., Bertino M.F., Strzelec K. (2018). Rigid polyurethane foams reinforced with industrial potato protein. Polym. Test..

[164] Członka S., Strąkowska A., Kairytė A. (2020). Effect of walnut shells and silanized walnut shells on the mechanical and thermal properties of rigid polyurethane foams. Polym. Test..

[165] Członka S., Strąkowska A., Strzelec K., Kairytė A., Kremensas A. (2020). Bio-based polyurethane composite foams with improved mechanical, thermal, and antibacterial properties. Materials.

[166] Członka S., Strąkowska A. (2020). Rigid polyurethane foams based on bio-polyol and additionally reinforced with silanized and acetylated walnut shells for the synthesis of environmentally friendly insulating materials. Materials.

[167] Członka S., Strąkowska A., Kairytė A. (2020). The impact of hemp shives impregnated with selected plant oils on mechanical, thermal, and insulating properties of polyurethane composite foams. Materials.

[168] Hejna A., Olszewski A., Zedler Ł., Kosmela P., Formela K. (2021). The impact of ground tire rubber oxidation with H_2_O_2_ and KMnO_4_ on the structure and performance of flexible polyurethane/ground tire rubber composite foams. Materials.

[169] Kosmela P., Olszewski A., Zedler Ł., Burger P., Piasecki A., Formela K., Hejna A. (2021). Ground tire rubber filled flexible polyurethane foam—Effect of waste rubber treatment on composite performance. Materials.

[170] Członka S., Strąkowska A., Kairytė A., Kremensas A. (2020). Nutmeg filler as a natural compound for the production of polyurethane composite foams with antibacterial and anti-aging properties. Polym. Test..

[171] Paciorek-Sadowska J., Borowicz M., Isbrandt M. (2021). Effect of evening primrose (*Oenothera biennis*) oil cake on the properties of polyurethane/polyisocyanurate bio-composites. Int. J. Mol. Sci..

[172] Leszczyńska M., Ryszkowska J., Szczepkowski L., Kurańska M., Prociak A., Leszczyński M.K., Gloc M., Antos-Bielska M., Mizera K. (2020). Cooperative effect of rapeseed oil-based polyol and egg shells on the structure and properties of rigid polyurethane foams. Polym. Test..

[173] Kairytė A., Kremensas A., Vaitkus S., Członka S., Strąkowska A. (2020). Fire suppression and thermal behavior of biobased rigid polyurethane foam filled with biomass incineration waste ash. Polymers.

[174] Członka S., Strąkowska A., Pospiech P., Strzelec K. (2020). Effects of chemically treated eucalyptus fibers on mechanical, thermal and insulating properties of polyurethane composite foams. Materials.

[175] Reghunadhan A., Datta J., Jaroszewski M., Kalarikkal N., Thomas S. (2020). Polyurethane glycolysate from industrial waste recycling to develop low dielectric constant, thermally stable materials suitable for the electronics. Arab. J. Chem..

[176] Kemona A., Piotrowska M. (2020). Polyurethane recycling and disposal: Methods and prospects. Polymers.

[177] Heiran R., Ghaderian A., Reghunadhan A., Sedaghati F., Thomas S., Haghighi A.H. (2021). Glycolysis: An efficient route for recycling of end of life polyurethane foams. J. Polym. Res..

[178] Deng Y., Dewil R., Appels L., Ansart R., Baeyens J., Kang Q. (2021). Reviewing the thermo-chemical recycling of waste polyurethane foam. J. Environ. Manag..

[179] Jutrzenka Trzebiatowska P., Dzierbicka A., Kamińska N., Datta J. (2018). The influence of different glycerine purities on chemical recycling process of polyurethane waste and resulting semi-products. Polym. Int..

[180] Jutrzenka-Trzebiatowska P., Beneš H., Datta J. (2019). Evaluation of the glycerolysis process and valorisation of recovered polyol in polyurethane synthesis. React. Funct. Polym..

[181] Datta J., Pasternak S. (2005). Oligourethane glycols obtained in glycolysis of polyurethane foam as semi-finished products for cast urethane elastomers preparation. Polimery.

[182] Molero C., de Lucas A., Rodríguez J.F. (2009). Activities of octoate salts as novel catalysts for the transesterification of flexible polyurethane foams with diethylene glycol. Polym. Degrad. Stab..

[183] Njuguna J.K., Muchiri P., Mwema F.M., Karuri N.W., Herzog M., Dimitrov K. (2021). Determination of thermo-mechanical properties of recycled polyurethane from glycolysis polyol. Sci. Afr..

[184] Simón D., Borreguero A.M., de Lucas A., Rodríguez J.F. (2015). Glycolysis of viscoelastic flexible polyurethane foam wastes. Polym. Degrad. Stab..

[185] Zahedifar P., Pazdur L., Vande Velde C.M.L., Billen P. (2021). Multistage chemical recycling of polyurethanes and dicarbamates: A glycolysis–hydrolysis demonstration. Sustainability.

[186] del Amo J., Borreguero A.M., Ramos M.J., Rodríguez J.F. (2021). Glycolysis of polyurethanes composites containing nanosilica. Polymers.

[187] Simón D., de Lucas A., Rodríguez J.F., Borreguero A.M. (2016). Glycolysis of high resilience flexible polyurethane foams containing polyurethane dispersion polyol. Polym. Degrad. Stab..

[188] Simón D., Borreguero A.M., de Lucas A., Rodríguez J.F. (2014). Glycolysis of flexible polyurethane wastes containing polymeric polyols. Polym. Degrad. Stab..

[189] Liu Z., Zhang C., Shi Z., Yin L., Tian M. (2018). Tailoring vinylogous urethane chemistry for the cross-linked polybutadiene: Wide freedom design, multiple recycling methods, good shape memory behavior. Polymer.

[190] Imiela M., Anyszka R., Bieliński D.M., Masłowski M., Pędzich Z., Ziąbka M., Rybiński P., Syrek B. (2019). Effect of graphite and common rubber plasticizers on properties and performance of ceramizable styrene–butadiene rubber-based composites. J. Therm. Anal. Calorim..

[191] Marzec A., Szadkowski B., Rogowski J., Rybiński P., Maniukiewicz W. (2021). Novel eco-friendly hybrid pigment with improved stability as a multifunctional additive for elastomer composites with reduced flammability and pH sensing properties. Dyes Pigm..

[192] Paszkiewicz S., Irska I., Zubkiewicz A., Szymczyk A., Piesowicz E., Rozwadowski Z., Goracy K. (2021). Biobased thermoplastic elastomers: Structure-property relationship of poly (hexamethylene 2,5-furanodicarboxylate)-block-poly (tetrahydrofuran) copolymers prepared by melt polycondensation. Polymers.

[193] Reghunadhan A., Jibin K.P., Kaliyathan A.V., Velayudhan P., Strankowski M., Thomas S. (2021). Shape memory materials from rubbers. Materials.

[194] Sarkar P., Bhowmick A.K. (2018). Sustainable rubbers and rubber additives. J. Appl. Polym. Sci..

[195] Hassan A.A., Abbas A., Rasheed T., Bilal M., Iqbal H.M.N., Wang S. (2019). Development, influencing parameters and interactions of bioplasticizers: An environmentally friendlier alternative to petro industry-based sources. Sci. Total Environ..

[196] Dziemidkiewicz A., Anyszka R., Blume A., Maciejewska M. (2020). Reaction mechanism of halogenated rubber crosslinking using a novel environmentally friendly curing system. Polym. Test..

[197] Dominic C.D.M., Joseph R., Begum P.M.S., Joseph M., Padmanabhan D., Morris L.A., Kumar A.S., Formela K. (2020). Cellulose nanofibers isolated from the Cuscuta reflexa plant as a green reinforcement of natural rubber. Polymers.

[198] Greenough S., Dumont M.-J., Prasher S. (2021). The physicochemical properties of biochar and its applicability as a filler in rubber composites: A review. Mater. Today Commun..

[199] Bockstal L., Berchem T., Schmetz Q., Richel A. (2019). Devulcanisation and reclaiming of tires and rubber by physical and chemical processes: A review. J. Clean. Prod..

[200] Dierkes W.K., Dijkhuis K., Hoek H.V., Noordermeer J.W.M., Reuvekamp L.A.E.M., Saiwari S., Blume A. (2019). Designing of cradle-to-cradle loops for elastomer products. Plast. Rubber Compos..

[201] Markl E., Lackner M. (2020). Devulcanization technologies for recycling of tire-derived rubber: A review. Materials.

[202] Saputra R., Walvekar R., Khalid M., Mubarak N.M., Sillanpää M. (2021). Current progress in waste tire rubber devulcanization. Chemosphere.

[203] Formela K. (2021). Sustainable development of waste tires recycling technologies—Recent advances, challenges and future trends. Adv. Ind. Eng. Polym. Res..

[204] Chittella H., Yoon L.W., Ramarad S., Lai Z.-W. (2021). Rubber waste management: A review on methods, mechanism, and prospects. Polym. Degrad. Stab..

[205] Massarotto M., da Silva Crespo J., Zattera A.J., Zeni M. (2008). Characterization of ground SBR scraps from shoe industry. Mater. Res..

[206] Luna C.B.B., Araújo E.M., Siqueira D.D., de Souza Morais D.D., dos Santos Filho E.A., Fook M.V.L. (2020). Incorporation of a recycled rubber compound from the shoe industry in polystyrene: Effect of SBS compatibilizer content. J. Elastom. Plast..

[207] Tozzi K.A., Canto L.B., Scuracchio C.H. (2020). Reclaiming of vulcanized rubber foam waste from the shoe industry through solid-state shear extrusion and compounding with SBR. Macromol. Symp..

[208] Rajan V.V., Dierkes W.K., Joseph R., Noordermeer J.W.M. (2006). Recycling of NR based cured latex material reclaimed with 2,2′-dibenzamidodiphenyldisulphide in a truck tire tread compound. J. Appl. Polym. Sci..

[209] ALbiajawi M.I., Alkasawneh R.W., Mostafa S.A., Johari I., Embong R., Muthusamy K. (2021). Performance of sustainable concrete containing recycled latex gloves and silicone catheter under elevated temperature. J. King Saud. Univ. Eng. Sci..

[210] Polat K., Bursalı E.A. (2019). A promising strategy for the utilization of waste nitrile gloves: Cost-effective adsorbent synthesis. J. Mater. Cycles Waste. Manag..

[211] Pacheco-Torgal F., Ding Y.N., Jalali S. (2012). Properties and durability of concrete containing polymeric wastes (type rubber and polyethylene terephthalate bottles): An overview. Constr. Build. Mater..

[212] Liu L., Cai G., Zhang J., Liu X., Liu K. (2020). Evaluation of engineering properties and environmental effect of recycled waste tire-sand/soil in geotechnical engineering: A compressive review. Renew. Sustain. Energy Rev..

[213] Przydatek G., Budzik G., Janik M. (2022). Effectiveness of selected issues related to used tyre management in Poland. Environ. Sci. Pollut. Res..

[214] European Tyre and Rubber Manufacturers’ Association: End of Life Tyres Management—Europe—2019. https://www.etrma.org/wp-content/uploads/2021/05/20210520_ETRMA_PRESS-RELEASE_ELT-2019.pdf.

[215] Williams P.T., Besler S. (1995). Pyrolysis-thermogravimetric analysis of tyres and tyre components. Fuel.

[216] Zedler Ł., Kowalkowska-Zedler D., Vahabi H., Saeb M.R., Colom X., Cañavate J., Wang S., Formela K. (2019). Preliminary investigation on auto-thermal extrusion of ground tire rubber. Materials.

[217] https://tyromer.com/product/.

[218] Ghavibazoo A., Abdelrahman M. (2013). Composition analysis of crumb rubber during interaction with asphalt and effect on properties of binder. Int. J. Pavement Eng..

[219] Thodesen C., Shatanawi K., Amirkhanian S. (2009). Effect of crumb rubber characteristics on crumb rubber modified (CRM) binder viscosity. Constr. Build. Mater..

[220] Liu S., Cao W., Fang J., Shang S. (2009). Variance analysis and performance evaluation of different crumb rubber modified (CRM) asphalt. Constr. Build. Mater..

[221] Rajalingham P., Sharpe J., Baker W.E. (1993). Ground rubber tire/thermoplastic composites: Effect of different ground rubber tires. Rubber Chem. Technol..

[222] Colom X., Marín-Genescà M., Mujal R., Formela K., Cañavate J. (2018). Structural and physico-mechanical properties of natural rubber/GTR composites devulcanized by microwaves: Influence of GTR source and irradiation time. J. Compos. Mater..

[223] Pehlken A., Müller D.H. (2009). Using information of the separation process of recycling scrap tires for process modeling. Resour. Conserv. Recy..

[224] Adhikari K., Das A., Sinha T., Saha P., Kuk Kim J., Kim J.K., Saha P., Thomas S., Haponiuk J.T., Aswathi M.K. (2018). Chapter 1: Grinding of waste rubber. Rubber Recycling: Challenges and Developments.

[225] Shen J., Amirkhanian S., Xiao F., Tang B. (2009). Influence of surface area and size of crumb rubber on high temperature properties of crumb rubber modified binders. Constr. Build. Mater..

[226] Product catalog 2021—Granulates. https://www.recykl.pl.

[227] Seghar S., Asaro L., Rolland-Monnet M., Aït Hocine N. (2019). Thermo-mechanical devulcanization and recycling of rubber industry waste. Resour. Conserv. Recycl..

[228] Isayev A.I., Liang T., Lewis T.M. (2014). Effect of particle size on ultrasonic devulcanization of tire rubber in twin-screw extruder. Rubber Chem. Technol..

[229] Tao G., He Q., Xia Y., Jia G., Yang H., Ma W. (2013). The effect of devulcanization level on mechanical properties of reclaimed rubber by thermal-mechanical shearing devulcanization. J. Appl. Polym. Sci..

[230] Yazdani H., Karrabi M., Ghasmi I., Azizi H., Bakhshandeh G.R. (2011). Devulcanization of waste tires using a twin-screw extruder: The effects of processing conditions. J. Vinyl Addit. Technol..

[231] de Sousa F.D.B., Zanchet A., Scuracchio C.H. (2017). Influence of reversion in compounds containing recycled natural rubber: In search of sustainable processing. J. Appl. Polym. Sci..

[232] de Sousa F.D.B., Ornaghi Júnior H.L. (2020). From devulcanization of ground tire rubber by microwaves to revulcanization: A revulcanization kinetic approach using a simple prediction model. ACS Sustain. Chem. Eng..

[233] de Sousa F.D.B., Zanchet A., Ornaghi H.L., Ornaghi F.G. (2019). Revulcanization kinetics of waste tire rubber devulcanized by microwaves: Challenges in getting recycled tire rubber for technical application. ACS Sustain. Chem. Eng..

[234] Sripornsawat B., Saiwari S., Pichaiyut S., Nakason C. (2016). Influence of ground tire rubber devulcanization conditions on properties of its thermoplastic vulcanizate blends with copolyester. Eur. Polym. J..

[235] Garcia P.S., Gouveia R.F., Maia J.M., Scuracchio C.H., Cruz S.A. (2018). 2D and 3D imaging of the deformation behavior of partially devulcanized rubber/polypropylene blends. Express Polym. Lett..

[236] Xu G., Kong P., Yu Y., Yang J., Zhu M., Chen X. (2022). Rheological properties of rubber modified asphalt as function of waste tire rubber reclaiming degree. J. Clean. Prod..

[237] Kroll L., Hoyer S. (2019). Zero-waste production: Technology for the in-house recycling of technical elastomers. Procedia Manuf..

[238] Dobrotă D., Dobrotă G. (2018). An innovative method in the regeneration of waste rubber and the sustainable development. J. Clean. Prod..

[239] Holka H., Wełnowski J. (2011). Non-conventional method of tires utilization. Inżynieria Masz..

[240] Wang Z., Kang Y., Cheng Y. (2017). Multiresponse optimization of process parameters in water jet pulverization via response surface methodology. Int. J. Precis. Eng. Manuf..

[241] Wang Z., Kang Y., Wang Z. (2017). Pulverization of end-of-life tires by ultra-high pressure water jet process. J. Polym. Eng..

[242] Holka H., Jarzyna T. (2017). Recycling of car tires by means of Waterjet technologies. AIP Conf. Proc..

[243] Bowles A.J., Fowler G.D., O’Sullivan C., Parker K. (2020). Sustainable rubber recycling from waste tyres by waterjet: A novel mechanistic and practical analysis. Sustain. Mater. Technol..

[244] Zefeng W., Yong K., Zhao W., Yi C. (2018). Recycling waste tire rubber by water jet pulverization: Powder characteristics and reinforcing performance in natural rubber composites. J. Polym. Eng..

[245] Park J.-M., An J.-Y., Bang D., Kim B.-S., Oh M.-H. (2014). Characteristics studies of waste tire rubber powders using the different grinding methods. J. Korean Inst. Resour. Recycl..

[246] Hrdlička Z., Brejcha J., Šubrt J., Vrtiška D., Malinová L., Čadek D., Kadeřábková A. (2021). Ground tyre rubber produced via ambient, cryogenic, and waterjet milling: The influence of milling method and particle size on the properties of SBR/NR/BR compounds for agricultural tyre treads. Plast. Rubber Compos..

[247] Li X., Xu X., Liu Z. (2020). Cryogenic grinding performance of scrap tire rubber by devulcanization treatment with scCO_2_. Powder Technol..

[248] Wang Z., Zeng D. (2021). Preparation of devulcanized ground tire rubber with supercritical carbon dioxide jet pulverization. Mater. Lett..

[249] Scuracchio C.H., Bretas R.E.S., Isayev A.I. (2004). Blends of PS with SBR devulcanized by ultrasound: Rheology and morphology. J. Elastom. Plast..

[250] de Sousa F.D.B., Zanchet A., Scuracchio C.H. (2019). From devulcanization to revulcanization: Challenges in getting recycled tire rubber for technical applications. ACS Sustain. Chem. Eng..

[251] Gągol M., Boczkaj G., Haponiuk J., Formela K. (2015). Investigation of volatile low molecular weight compounds formed during continuous reclaiming of ground tire rubber. Polym. Degrad. Stab..

[252] Simon D.A., Bárány T. (2021). Effective thermomechanical devulcanization of ground tire rubber with a co-rotating twin-screw extruder. Polym. Degrad. Stab..

[253] Shi J., Jiang K., Ren D., Zou H., Wang Y., Lv X., Zhang L. (2013). Structure and performance of reclaimed rubber obtained by different methods. J. Appl. Polym. Sci..

[254] Saiwari S., Dierkes W.K., Noordermeer J.W.M. (2014). Comparative investigation of the devulcanization parameters of tire rubbers. Rubber Chem. Technol..

[255] Wiśniewska P., Zedler Ł., Formela K. (2021). Processing, performance properties, and storage stability of ground tire rubber modi-fied by dicumyl peroxide and ethylene-vinyl acetate copolymers. Polymers.

[256] Formela K., Cysewska M., Haponiuk J. (2016). Thermomechanical reclaiming of ground tire rubber via extrusion at low temperature: Efficiency and limits. J. Vinyl Addit. Technol..

[257] Formela K., Klein M., Colom X., Saeb M.R. (2016). Investigating the combined impact of plasticizer and shear force on the efficiency of low temperature reclaiming of ground tire rubber (GTR). Polym. Degrad. Stab..

[258] Zedler Ł., Klein M., Saeb M.R., Colom X., Cañavate J., Formela K. (2018). Synergistic effects of bitumen plasticization and microwave treatment on short-term devulcanization of ground tire rubber. Polymers.

[259] Nunes A.T., dos Santos R.E., Pereira J.S., Barbosa R., Ambrósio J.D. (2018). Characterization of waste tire rubber devulcanized in twin-screw extruder with thermoplastics. Prog. Rubber Plast. Recycl. Technol..

[260] Barbosa R., Ambrósio J.D. (2019). Devulcanization of natural rubber compounds by extrusion using thermoplastics and characterization of revulcanized compounds. J. Polym. Res..

[261] Zedler Ł., Kowalkowska-Zedler D., Colom X., Cañavate J., Saeb M.R., Formela K. (2020). Reactive sintering of ground tire rubber (GTR) modified by a trans-polyoctenamer rubber and curing additives. Polymers.

[262] Formela K., Hejna A., Zedler Ł., Colom X., Cañavate J. (2019). Microwave treatment in waste rubber recycling—recent advances and limitations. Express Polym. Lett..

[263] Paulo G.D., Hirayama D., Saron C. (2012). Microwave devulcanization of waste rubber with inorganic salts and nitric acid. Adv. Mater. Res..

[264] Seghar S., Aït Hocine N., Mittal V., Azem S., Al-Zohbi F., Schmaltz B., Poirot N. (2015). Devulcanization of styrene butadiene rubber by microwave energy: Effect of the presence of ionic liquid. Express Polym. Lett..

[265] Poyraz S., Liu Z., Liu Y., Zhang X. (2013). Devulcanization of scrap ground tire rubber and successive carbon nanotube growth by microwave irradiation. Curr. Org. Chem..

[266] Jia L.-C., Li Y.-K., Yan D.-X. (2017). Flexible and efficient electromagnetic interference shielding materials from ground tire rubber. Carbon.

[267] Liu J., Liu P., Zhang X., Lu P., Zhang X., Zhang M. (2016). Fabrication of magnetic rubber composites by recycling waste rubber powders via a microwave-assisted in situ surface modification and semi-devulcanization process. Chem. Eng. J..

[268] Zhang X., Zhu X., Liang M., Lu C. (2009). Improvement of the properties of ground tire rubber (GTR)-filled nitrile rubber vulcanizates through plasma surface modification of GTR powder. J. Appl. Polym. Sci..

[269] Cheng X., Chen H., Huang S., Li Z., Guo X. (2012). Improvement of the properties of plasma-modified ground tire rubber filled cement paste. J. Appl. Polym. Sci..

[270] Nisticò R., Lavagna L., Boot E.A., Ivanchenko P., Lorusso M., Bosia F., Pugno N.M., D’Angelo D., Pavese M. (2021). Improving rubber concrete strength and toughness by plasma-induced end-of-life tire rubber surface modification. Plasma Process Polym..

[271] Rungrodnimitchai S., Kotatha D. (2015). Chemically modified ground tire rubber as fluoride ions adsorbents. Chem. Eng. J..

[272] Araujo-Morera J., Verdugo-Manzanares R., González S., Verdejo R., Lopez-Manchado M.A., Hernández Santana M. (2021). On the use of mechano-chemically modified ground tire rubber (GTR) as recycled and sustainable filler in styrene-butadiene rubber (SBR) composites. J. Compos. Sci..

[273] Klajn K., Gozdek T., Bieliński D.M., Siciński M., Zarzecka-Napierała M., Pędzich Z. (2021). SBR vulcanizates filled with modified ground tire rubber. Materials.

[274] Ma L., Zhang Z., Peng Z., Formela K., Wang S. (2021). Dynamic mechanical properties and flexing fatigue resistance of tire sidewall rubber as function of waste tire rubber reclaiming degree. J. Appl. Polym. Sci..

[275] Shi J., Zou H., Ding L., Li X., Jiang K., Chen T., Zhang X., Zhang L., Ren D. (2014). Continuous production of liquid reclaimed rubber from ground tire rubber and its application as reactive polymeric plasticizer. Polym. Degrad. Stab..

[276] Formela K., Korol J., Saeb M.R. (2015). Interfacially modified LDPE/GTR composites with non-polar elastomers: From microstructure to macro-behavior. Polym. Test..

[277] Formela K., Sulkowski M., Saeb M.R., Colom X., Haponiuk J.T. (2016). Assessment of microstructure, physical and thermal properties of bitumen modified with LDPE/GTR/elastomer ternary blends. Constr. Build. Mater..

[278] Boczkowska A. (2012). Advanced Elastomers. Technology, Properties and Applications.

[279] Utrera-Barrios S., Verdejo R., Angel López-Manchado M., Hernández Santana M. (2020). Evolution of self-healing elastomers, from extrinsic to combined intrinsic mechanisms: A review. Mater. Horiz..

[280] Ma Z., Li H., Jing X., Liu Y., Mia H.-Y. (2021). Recent advancements in self-healing composite elastomers for flexible strain sensors: Materials, healing systems, and features. Sens. Actuator A Phys..

[281] Hernández Santana M., Huete M., Lameda P., Araujo J., Verdejo R., López-Manchado M.A. (2018). Design of a new generation of sustainable SBR compounds with good trade-off between mechanical properties and self-healing ability. Eur. Polym. J..

[282] Araujo-Morera J., Hernández Santana M., Verdejo R., López-Manchado M.A. (2019). Giving a second opportunity to tire waste: An alternative path for the development of sustainable self-healing styrene-butadiene rubber compounds overcoming the magic triangle of tires. Polymers.

[283] Alonso Pastor L.E., Núñez Carrero K.C., Araujo-Morera J., Hernández Santana M., Pastor J.M. (2022). Setting relationships between structure and devulcanization of ground tire rubber and their effect on self-healing elastomers. Polymers.

[284] Toczek K., Lipińska M., Pietrasik J. (2021). Smart TPE materials based on recycled rubber shred. Materials.

[285] Zedler Ł., Burger P., Wang S., Formela K. (2020). Ground tire rubber modified by ethylene-vinyl acetate copolymer: Processing, physico-mechanical properties, volatile organic compounds emission and recycling possibility. Materials.

[286] Domingues J., Marques T., Mateus A., Carreira P., Malça C. (2017). An additive manufacturing solution to produce big green parts from tires and recycled plastics. Procedia Manuf..

[287] Alkadi F., Lee J., Yeo J.S., Hwang S.-H., Choi J.-W. (2019). 3D printing of ground tire rubber composites. Int. J. Precis. Eng. Manuf.-Green Tech..

[288] Toncheva A., Brison L., Dubois P., Laoutid F. (2021). Recycled tire rubber in additive manufacturing: Selective laser sintering for polymer-ground rubber composites. Appl. Sci..

[289] Laoutid F., Lafqir S., Toncheva A., Dubois P. (2021). Valorization of recycled tire rubber for 3D printing of ABS- and TPO-based composites. Materials.

